# Multi‐omics profiling identifies *TNFRSF18* as a novel marker of exhausted CD8⁺ T cells and reveals tumour‐immune dynamics in colorectal cancer

**DOI:** 10.1002/ctm2.70425

**Published:** 2025-08-06

**Authors:** Tengfei Jia, Yingxi Guo, Xin meng Cheng, Zeyang Zhou, Xiaojiang Xu, Hebin Liu, Xiaodong Yang

**Affiliations:** ^1^ Institutes of Biology and Medical Sciences Soochow University Suzhou China; ^2^ Department of Gastrointestinal Surgery The Second Affiliated Hospital of Soochow University Suzhou China; ^3^ Tulane University School of Medicine New Orleans Louisiana USA; ^4^ Cancer Institute Suzhou Medical College, Soochow University Suzhou China; ^5^ National Center of Technology Innovation for Biopharmaceuticals Suzhou China

**Keywords:** colorectal cancer, singlecell RNA sequencing, spatial transcriptomics, T cell exhaustion, TNFRSF18 (GITR), TNM stage

## Abstract

**Background:**

Colorectal cancer (CRC) ranks among the most prevalent malignant tumours of the digestive system globally and is associated with unfavourable survival outcomes. The exhaustion of CD8⁺ T cells serves a crucial role in facilitating tumour immune escape. Yet, the dynamic evolution of CD8⁺ T cell exhaustion and its impact on clinical prognosis across TNM (tumour‐node‐metastasis) stages in CRC remains incompletely characterized.

**Methods:**

Tumour and adjacent tissues (20 samples total) from 6 CRC patients spanning diverse TNM stages were analyzed using integrated single‐cell transcriptomic profiling (scRNA‐seq), single‐cell T cell receptor/B cell receptor sequencing (scVDJ‐seq), and spatial transcriptomics. T cell exhaustion markers, immune clonality, gene expression profiles, and the spatial distribution of both tumour cells and immune cells were systematically profiled. Functional enrichment and intercellular communication analyses were conducted. Key findings were validated using immunofluorescence and public datasets.

**Results:**

Our results illustrate how advancing TNM stages in CRC shape CD8⁺ T cell exhaustion through divergent TNFRSF18/CXCL13 dynamics and ribosomal stemness. *TNFRSF18* expression was notably higher in T cells infiltrating tumour tissues relative to their counterparts in adjacent non‐tumorous areas, with high‐expressing CD8⁺ T cells exhibiting marked exhaustion features. During CRC progression, TNM‐stage‐driven remodelling of the tumour microenvironment (TME) induced progressive CD8⁺ T cell exhaustion marked by declining *TNFRSF18* and rising *CXCL13* expression in tumour‐infiltrating T cells elevation of both markers in the tumour compared with adjacent tissues. Moreover, we show that tumour cells displayed elevated expression of stemness‐associated ribosomal genes (*RPS7*, *RPL8*, *RPL30*), peaking at stage T4, which correlated with poor prognosis and immune escape.

**Conclusions:**

This integrative multi‐omics study uncovers CD8⁺ T cell exhaustion dynamics and ribosomal stemness‐mediated immune evasion across CRC progression. *CXCL13*, *TNFRSF18*, and ribosomal proteins (*RPS7*/*RPL8*/*RPL30*) are identified as novel biomarkers with direct prognostic value and therapeutic relevance, providing therapeutic targets for precision immunotherapy in CRC.

**Key points:**

1. Multi‐omics analysis reveals dynamic CD8^+^ T cell exhaustion patterns across CRC samples with different TNM stages.
2. TNFRSF18 is highly expressed in exhausted tumour‐infiltrating CD8^+^ T cells and declines with disease progression.
3. Ribosomal stemness in tumour cells promotes immune evasion by impairing TNF‐mediated CD8^+^ T cell function.

## Graphical abstract

Schematic model for TNFRSF18 decline fuels stemness‐driven immune evasion in colorectal cancer. Tumour cells with upregulated stemness‐related ribosomal proteins (RPS7/RPL30/RPL8) evade TNF‐mediated clearance. This TNFRSF18‐stemness axis forms a feed‐forward loop that promotes immune escape, representing a potential therapeutic target for CRC.

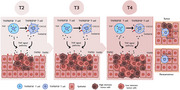

## 1 INTRODUCTION

Colorectal cancer (CRC) stands among the most prevalent malignancies affecting the gastrointestinal tract worldwide and remains a major contributor to global cancer‐related morbidity and mortality.^1^ Although notable progress has been made in surgical procedures, chemotherapeutic strategies, and targeted therapeutic approaches, the prognosis for CRC patients—particularly those diagnosed at advanced or metastatic stages—remains unsatisfactory,^2^ particularly for those with advanced or metastatic disease. Therefore, a deeper understanding of the tumour microenvironment (TME), with particular emphasis on the dynamic alterations within the immune landscape throughout CRC initiation and progression, is essential for improving diagnosis, treatment, and prognostic evaluation.^3^, ^4^, ^5^ Among the various immunosuppressive mechanisms within the TME, CD8⁺ T cell exhaustion is a key mechanism facilitating tumour immune evasion, and the discovery of its markers (e.g., PD‐1 and LAG‐3) has driven the advancement of immune checkpoint inhibitors; however, most patients are still challenged by insufficient response to therapy.^3^, ^4^

The tumour‐node‐metastasis (TNM) staging is an important clinical standard for assessing tumour infiltration, lymph node metastasis, and distant metastasis in CRC patients, reflecting anatomical disease extent and metastatic burden.^5^ However, this system provides limited information regarding the functional immunological landscape within the tumour, which evolves dynamically during CRC progression. A systematic understanding of how immune cell composition, function, and cellular crosstalk change across TNM stages remains elusive, hindering the development of stage‐specific immunotherapeutic strategies. Recent advancements in single‐cell resolution analysis, including scRNA‐seq, scVDJ‐seq, and spatial transcriptomics (ST), have introduced powerful tools for delineating the cellular heterogeneity and spatial architecture of the TME, while enabling high‐resolution analysis of immune cell dynamics within tumour tissues.^6^, ^7^, ^8^ Applying these integrative multi‐omics approaches holds promise for mapping the evolving immune landscape across CRC progression and uncovering the molecular circuits linking tumour cell biology and immune dysfunction.

Colorectal cancer progression is critically fueled by a distinct subset of tumour‐initiating cells (TICs) endowed with stem cell–like characteristics, characterized by self‐renewal capacity, therapy resistance, and metastatic potential,^9^ which correlates strongly with poor prognosis and immune evasion.^10^ Ribosomal dysregulation sustains TIC states, where aberrant expression of ribosomal proteins (RPS/RPL family) reprograms translational control to favour pro‐survival and stemness programs, enabling TICs to resist immune surveillance.^11^ Cancer cell stemness refers to the ability of a minority of tumour cells to acquire properties typically associated with normal stem cells, such as self‐renewal, multipotent differentiation potential, and resistance to therapeutic interventions. These stem‐like tumour cell subsets, often referred to as cancer stem cells (CSCs), are crucial contributors to tumour onset, malignant progression, and metastatic dissemination; elucidating the mechanisms that regulate cancer cell stemness is essential for developing targeted therapies to eliminate CSCs and enhance patient prognosis.

In this work, we conducted a comprehensive multi‐omics analysis on tumour tissues and matched adjacent non‐tumorous samples collected from colorectal cancer (CRC) patients representing various TNM stages. By combining scRNA‐seq and stRNA‐seq technologies, we revealed stage‐specific gene expression characteristics of T cells, uncovered pivotal exhaustion‐associated markers in CD8⁺ T cell populations, including *TNFRSF18* and *CXCL13*,^9^, ^12^, ^13^, ^14^, ^15^ and explored the communication networks between immune cells and tumour cells.^16^ The discovery of stem cell‐related genes (*RPS7*, etc.) and *TNFRSF18*/*CXCL13* provides a new direction for prognostic assessment and targeted therapy. The integrated multi‐omics analysis provides a new paradigm for tumour heterogeneity research.

## 2 MATERIALS AND METHODS

### 2.1 Patients and tissue sample collection

This study was approved by the Ethics Committee of the Second Affiliated Hospital of Soochow University (JD‐LK‐2020‐004‐01). Six CRC patients provided written informed consent for participation in scRNA‐seq analysis. Patient selection was independent of sex. Primary CC, P (at least 2 cm from the tumour), LM, and LP (at least 2 cm from the tumour) tissues were collected post‐surgery. Preoperative chemotherapy and/or radiotherapy were administered to all patients. For spatial transcriptomics (ST) sequencing, formalin‐fixed paraffin‐embedded (FFPE) tissues from three CRC patients were prepared. Table S1 presents an overview of the clinical and demographic data for the patient cohort.

### 2.2 Tissue dissociation and preparation of single‐cell suspensions

Tissues were placed on wet ice in a Petri dish with 1× PBS (free of RNase, Ca, and Mg ions) and washed to remove contaminants. The tissue was cut into small pieces and treated with a dissociation solution containing collagenase IV, papain, and DNase I at 37°C for 20 min. The reaction was stopped with PBS containing 10% fetal bovine serum. Cells were pipetted, filtered, and centrifuged. Erythrocytes were lysed, and dead cells were removed using Dead Cell Removal MicroBeads and MS Columns. The remaining cells were washed with PBS (.04% BSA) and centrifuged. The final cell suspension was assessed for viability using trypan blue, ensuring >85% viability. Cell counting was performed using a hemocytometer or automated cell counter, with a concentration of 700–1200 cells/µL.

### 2.3 Construction of Chromium 10× Genomics libraries and subsequent sequencing

Single‐cell suspensions were loaded onto the 10× Chromium chip following the manufacturer's protocol for the Chromium Single‐Cell 3′ kit (V3), targeting the capture of approximately 8000 cells. Subsequent cDNA amplification and library preparation were conducted according to standard procedures. Libraries were sequenced on an Illumina NovaSeq 6000 platform using paired‐end 150 bp reads, with a minimum sequencing depth of 20 000 reads per cell.

### 2.4 Spatial transcriptome sequencing process

CRC tissue sections were mounted on chilled Visium Tissue Optimization Slides and Visium Spatial Gene Expression Slides (10× Genomics). Following this, samples were fixed in cold methanol and stained according to the manufacturer's protocols for Visium Spatial Gene Expression or Tissue Optimization. Based on tissue optimization‐time course experiments, an incubation time of 18 min was selected as optimal. Library loading (300 pM) and sequencing (approximately 250–400 million read pairs per sample) were performed on an Illumina NovaSeq 6000 system using the NovaSeq S4 Reagent Kit (200 cycles, Illumina), following the Visium Spatial Gene Expression user manual.

### 2.5 Sequencing process for single‐cell immunobanking experiments

10× Genomic Single‐Cell Immune Repertoire sequencing is a microfluidic platform based on GemCode technology. Magnetic beads carrying barcodes and primers are encapsulated with individual cells inside oil droplets. Within each droplet, the gel beads dissolve and cells undergo lysis, releasing mRNA, which is then reverse‐transcribed to produce barcoded cDNA for sequencing. After the liquid–oil layer is disrupted, the cDNA is divided into two parts. Then, libraries for gene expression and immune repertoire are constructed. The V(D)J sequences of TCR or BCR are enriched by nested PCR primers designed in the C regions of TCR, BCR, or IG. Then, the Illumina NovaSeq 6000 sequencing platform (paired‐end sequencing, 150 bp) is used for library sequencing.

### 2.6 Preprocessing, dimensionality reduction, and clustering of scRNA‐seq and spatial transcriptomics data

The sequencing reads were aligned to the human reference genome GRCh38 using CellRanger (spaceranger for spatial data).^17^ All libraries were subsequently combined to correct for batch effects and normalize sequencing depth. An expression matrix, with genes as rows and cells as columns, was created and imported into the R package Seurat.^18^ Low‐quality cells and genes were then filtered out for further analysis. Cell quality control was performed from five perspectives: number of transcripts detected in each cell, number of genes, percentage of mitochondrial genes, percentage of ribosomal genes, and percentage of erythrocyte genes. The default cell filtering metrics were as follows: cells that simultaneously met 200 < gene count < 10 000, mitochondrial gene percentage <10%, and erythrocyte marker gene percentage <10%, and possible double‐cells were removed with Scrublet.^19^

Spatial transcriptomics data QC conditions: QC was performed from five perspectives: the number of UMIs detected by each Spot, the number of genes, the percentage of mitochondrial genes, the percentage of ribosomal genes, and the percentage of erythrocyte genes. The default Spot filtering index is: the Spot with gene numbers greater than or equal to 200 are selected to enter the downstream analysis. The first 30 PCA principal components were selected, and the PCA space was used to construct a nearest‐neighbour KNN graph based on the Euclidean distance, and then the cell clusters were clustered using the Louvain Modularity optimization algorithm, in which the RESOLUTION was set to .9. Dimensionality reduction: The first 30 PCA principal components were selected, and the cell clusters were clustered using tSNE^20^ and UMAP.^21^ Two‐dimensional reduction methods were employed to visualize the clustering of the single‐cell population. Batch effect removal is performed via the RunHarmony function in the Harmony package.^22^

### 2.7 Unsupervised cell clustering and annotation

Cells were annotated based on differentially expressed genes for each lineage, referencing published articles and related databases. Major cell types included epithelial, fibroblast, myeloid, plasma, B, endothelial, NK, MAST, neuroglial, and T cells.^23^ Subclusters were identified through a second round of UMAP clustering, and differentially expressed genes for each subcluster were identified through Seurat's FindAllMarkers function.

### 2.8 Monocle pseudotime analysis

Normalized single‐cell transcriptome expression matrices were imported into Monocle.^24^, ^25^, ^26^ Cell type annotations and experimental conditions were provided via metadata files. The select gene function was used to screen for highly variable genes associated with changes in cell state. These genes were used for subsequent downscaling and trajectory construction. The proposed temporal values were assigned to each cell by the order cells function. Apply DDRTree, which learns the structure of the dataset's manifold as a developmental trajectory and sorts the cells onto that manifold using the computed proposed chronology starting from a given root cell. The results of the trajectory are presented using Monocle's built‐in visualization tools.

### 2.9 GSEA enrichment analysis

Sene set enrichment analysis (GSEA) was performed using the clusterProfiler (RRID: SCR_016884) R package.^27^, ^28^, ^29^, ^30^ A ranked gene list was generated based on Log2FC, and gene sets were obtained from the Molecular Signatures Database (MSigDB). In this study, C2: curated gene sets were used, and the GSEA results were visualized by the ggplot2 (RRID: SCR_014601) package to plot the enrichment results.

### 2.10 Cell–cell interaction analysis

Single‐cell transcriptome data were input into the CellChat^31^ tool as a normalized expression matrix. We performed initial quality control and normalization of the data, including filtering of low‐quality cells, normalization of gene expression values and screening of highly variable genes. CellChat objects were created using the createCellChat function, intercellular communication networks were inferred from the ligand–receptor interactions database, and Identify Over Expressed Interactions functions were used to screen for overexpressed genes and ligand–receptor pairs. The communication probabilities were calculated using the compute Commun Prob function to screen for significant communication pairs. Subsequently, key signalling pathways were identified using the Compute Commun Prob Pathway function. To demonstrate the cellular communication network, we used the functions netVisual_circle and netVisual_heatmap.

### 2.11 TCGA survival analysis

Survival analysis was conducted using the Kaplan–Meier plotter (https://kmplot.com/analysis/),^32^ selecting the colorectal cancer module and parameters such as RFS, OS, and PPS. Relevant genes were input for prognosis analysis.

### 2.12 Spatial transcriptomics cell type decomposition analysis

Robust cell type decomposition (RCTD)^33^ was used to infer cell types and their relative abundances in spatial transcriptomics data. This tool infers the cell type and its relative abundance at each spatial location by mapping single‐cell transcriptome data to spatial transcriptome data. Single‐cell data and spatial transcriptome data are formatted into the required input format for RCTD using Seurat. The single‐cell transcriptome data are used as reference data to provide the expression profile of the cell type. The inferred spatial distribution of cell types was further validated by combining the expression patterns of key marker genes.

### 2.13 Immunofluorescence staining

Colorectal cancer (CRC) and adjacent tissues were collected at specific intervals, and fresh frozen sections were prepared. These sections were stored at −80°C after being embedded in Tissue‐Tek O.C.T compound (Sakura). We fixed the sections with 4% paraformaldehyde. After diluting primary antibodies with 1:500 in PHT and incubating at 4°C overnight, the secondary antibodies were diluted 1:1000 in PHT and incubated in the dark for 2.5 h. Hoechst staining was applied, and slides were analyzed using a Nikon DUX‐VB confocal microscope. Fluorescence intensity was measured with ImageJ (RRID: SCR_003070).

### 2.14 Malignant tumour cell identification

Copy number variation (CNV) in single‐cell transcriptome data was detected using the CopyKAT (RRID: SCR_024512) R package^34^ to distinguish tumour cells from normal cells. The expression matrix is in the form of genes as rows and barcodes as columns. Tumour cell subpopulations were identified based on CNV results.

### 2.15 Stemness analysis

CytoTRACE^35^ was used to infer cell differentiation potential and developmental trajectories based on gene expression and expression patterns. The input data required for CytoTRACE analysis is a normalized single‐cell gene expression matrix, where rows represent genes and columns represent individual cells. It has been subjected to routine quality control processes (e.g., removal of low‐quality cells and low‐expressed genes). The list of significant stemness‐related genes was extracted from the CytoTRACE output. Visualization was performed using CytoTRACE built‐in functions. The stemness score is performed via Seurat's AddModuleScore() function.^4^

### 2.16 Gene overexpression

*TNFRSF18* overexpression in Jurkat cells was established by electroporation. We constructed a pLVX vector carrying the *TNFRSF18* gene efficient expression in Jurkat cells. First, the logarithmic growth phase Jurkat cells were collected and pelleted by low‐temperature centrifugation (1000 rpm, 3 min). Then adjusted the cell concentration to 2.5 × 10^6^ cells/mL. Plasmid DNA (2 µg) was mixed with cell suspension (400 µL). Electroporation was performed using the JURKAT program on the Lonza Amaxa Nucleofector II Device with a 2 mm electroporation cuvette. The 37°C pre‐warmed RPMI‐1640 medium was immediately added to the cuvette after electroporation, and then the cells were transferred to a six‐well plate to facilitate recovery. At 48 h post‐transfection, *TNFRSF18* overexpression was confirmed by Western blot analysis.

### 2.17 RT‐qPCR

RT‐qPCR was performed according to the previous publication.^36^ The TRIzol Reagent (Sigma‐Aldrich, T9424) was used to isolate total RNA from tissues or cells. We used Hifair II 1st Strand cDNA Synthesis Kit (Yeasen, 11119ES60) to synthesizing first‐strand cDNA. Primers were obtained from Sangon Biotech, with sequences available in supplementary material 10. Then we performed qPCR on the QuantStudio Design and Analysis System (Applied Biosystems) with the Hieff Quantitative PCR SYBR Green Master Mix (Yeasen, 11184ES03). Using the ΔΔCt method to determine relative expression levels, normalized using a housekeeping gene.

### 2.18 Western blotting

Western blotting was performed according to a previous publication.^37^ Cells were lysed using a buffer containing 1% Triton X‐100 in 20 mM Tris‐HCl (pH 8.3) together with 150 mM NaCl, and an EDTA‐free protease inhibitor cocktail (Roche) was added. The lysates were separated by SDS‐PAGE, and then immunoblotting was performed with specialized primary antibodies. Detection was achieved using HRP‐ or IRDye‐conjugated secondary antibodies and visualized via enhanced chemiluminescence.

### 2.19 Statistics

Results were expressed as mean ± SEM in the analyses conducted using GraphPad Prism 10. Specifically, a two‐tailed unpaired Student's *t*‐test was employed for comparing the means of two groups.

## 3 RESULTS

### 3.1 Colorectal cancer sample collection and sequencing

**FIGURE 1:**
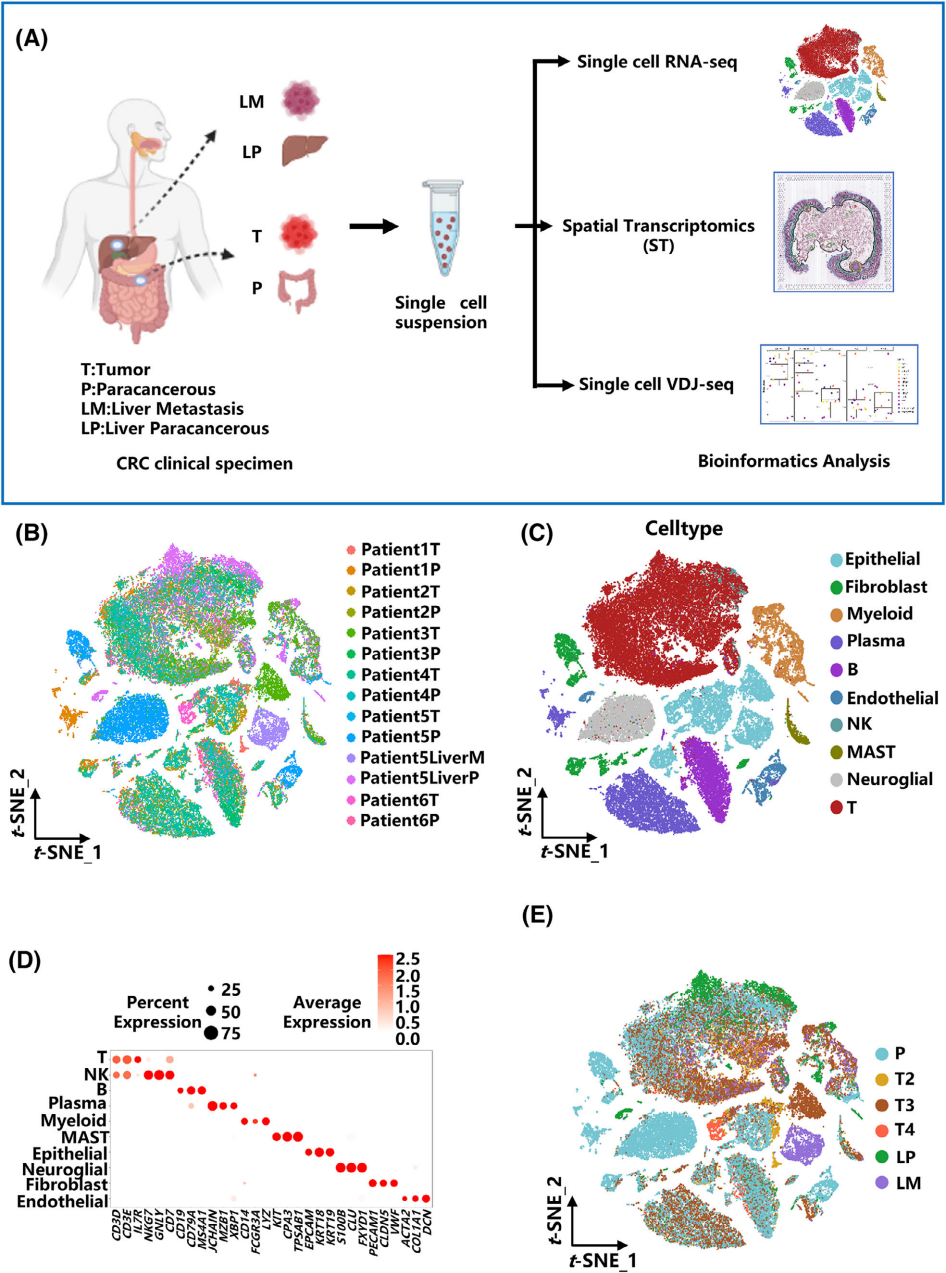
Multi‐omics sample processing, integration, and cellular atlas of the colorectal cancer microenvironment across TNM stages (A) Schematic overview of sample collection from CRC patients at varying TNM stages and matched adjacent non‐tumour tissues, followed by processing for integrated single‐cell RNA sequencing (scRNA‐seq), single‐cell VDJ sequencing (scVDJ‐seq), and spatial transcriptomics (stRNA‐seq) analysis. Twenty samples from each of the six patients were processed for sequencing and analyzed. (B) t‐SNE plot illustrating the cell distribution across different patients. Each dot represents an individual cell, with colour indicating the origin of the patient. (C) t‐SNE plot displaying the distribution of different cell types. Different colours represent different cell types. (D) The bubble chart shows marker genes used for definitive cell type identification. Bubble size represents the percentage of cells within a cluster expressing the gene. The colour intensity indicates the average normalized expression level. (E) TNM stage‐associated cellular landscape. t‐SNE visualization highlighting the distribution of cells grouped by their source tissue (tumour vs. paracancerous) and the clinical TNM stage of the originating tumour sample.

To investigate the dynamic evolution of the tumour microenvironment (TME) during colorectal cancer (CRC) progression and its association with TNM classification, aiming to uncover potential molecular targets with clinical therapeutic relevance. We comprehensively characterized the TME by integrating scRNA‐seq, scVDJ‐seq, and spatial transcriptomics (stRNA‐seq) analyses of surgically resected CRC tissues (Figure 1A). These samples included patient‐matched tumour tissue and paracancerous tissue. These multi‐omics data provide a further understanding of the mechanisms of tumour metastasis at work. In this study, paired tumour and adjacent non‐tumorous colonic tissues were obtained from the colonic tissues of six colorectal cancer patients (Patient 1–6). Among them, liver metastatic tumour tissue and paracancerous tissue samples were obtained from Patient 5 (two samples).

Single‐cell transcriptomic profiling was conducted on all 14 samples to profile the cellular landscape of CRC tissues. Additionally, spatial transcriptomic analysis was applied to tumour and adjacent non‐tumorous tissues collected from colonic samples of three patients at different clinical stages (Patient 2, 4 and 6) to investigate the dynamic changes associated with colorectal cancer progression across these stages (Table S1).

### 3.2 Single‐cell transcriptomic data processing, integration and cell type annotation

To ensure the quality of the data, cells exhibiting poor quality metrics or elevated mitochondrial gene content were removed. After excluding doublets by using Scrublet, 59 632 cells were retained. Data from all samples were pooled, normalized, and processed through principal component analysis (PCA). Potential batch effects were addressed by applying the Harmony algorithm for data integration, and the results were visualized via tSNE. These results revealed a high degree of overlap in the distribution of cells in clusters from different samples (Figure 1B), suggesting that cluster assignment was not caused by batch effects.

Subsequently, cell populations were classified according to canonical marker genes reported in previous literature and curated from the CellMarker database (Figure 1C). T cells (*CD3E*, *CD3D*, *IL7R*), B cells (*CD19*, *MS4A1*, *CD79A*), NK cells (*NKG7*, *GNLY*, *CD7*), plasma cells (*JCHAIN*, *MZB1*, *XBP1*), myeloid cells (*CD14*, *FCGR3A*, *LYZ*), epithelial cells (*EPCAM*, *KRT18*, *KRT19*), MAST cells (*KIT*, *CPA3*, *TPSAB1*), glial cells (*S100B*, *CLU*, *FXYD1*), fibroblasts (*PECAM1*, *CLDN5*, *VWF*), and endothelial cells (*ACTA2*, *COL1A1*, *DCN*) were identified (Figure 1D). Due to differences in patients and sampling sites, the number, types, and proportions of cells varied across the different samples. (Figure S1A; Table S2). In addition, the results of automatic annotation using SingleR^38^ were consistent with our manual annotations (Figure S1B).

### 3.3 Dynamic evolution of exhausted CD8⁺ T cell characteristics with tumour progression

**FIGURE 2:**
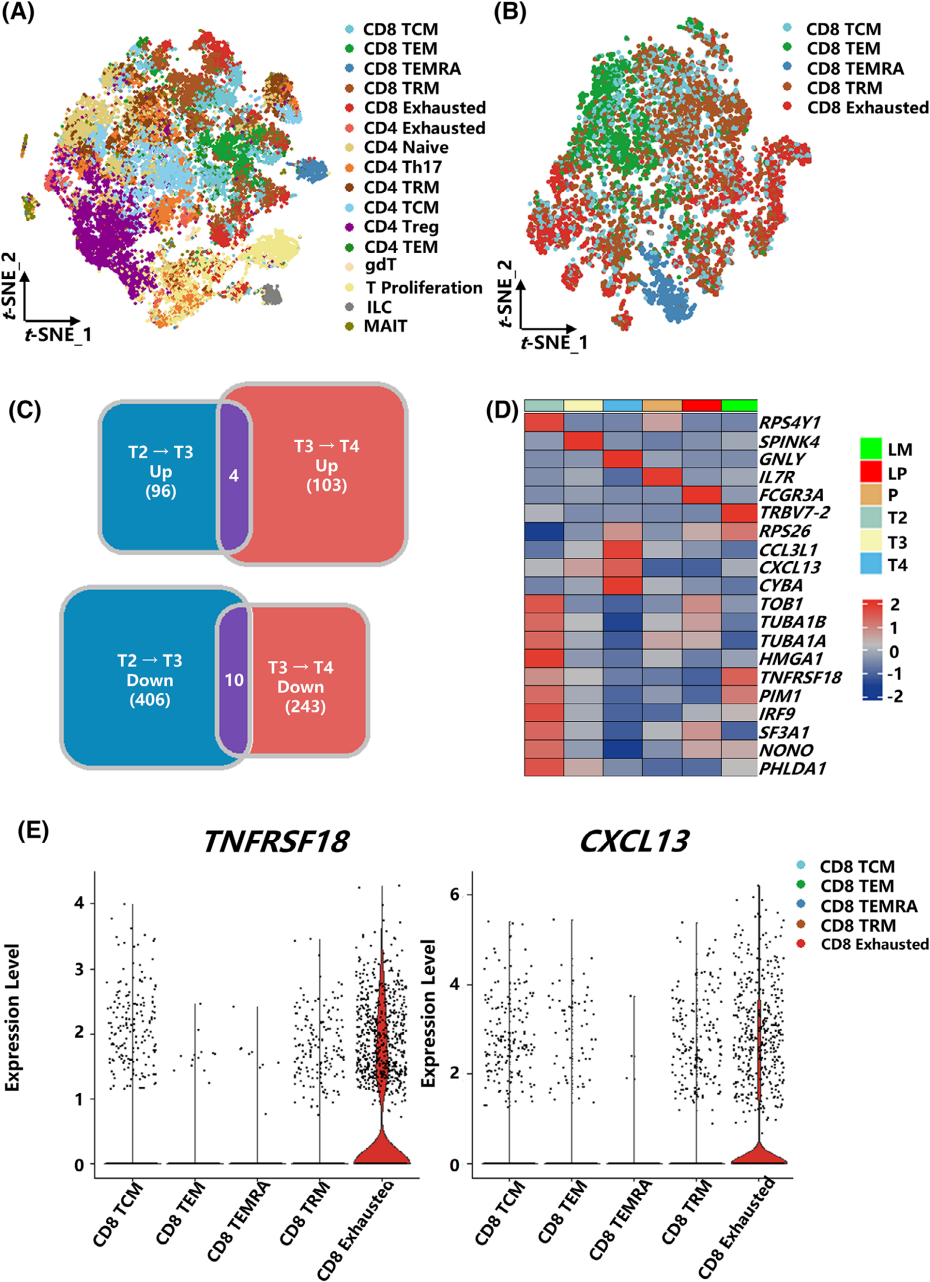
Stage‐dependent dynamics of T cell dysfunction and exhaustion markers in colorectal cancer progression. (A) t‐SNE plot illustrating the identified T‐cell subpopulations. (B) t‐SNE plot displaying the identified CD8⁺ T‐cell subpopulations. (C) The Venn diagram displays differentially expressed genes (DEGs) whose expression levels progressively increase or decrease with advancing clinical stage. Genes were selected based on a log_2_ fold change (log_2_FC) > .5 and a *p*‐value < .05. (D) Heatmap illustrating gene expression changes in CD8⁺ T cells across different clinical stages. Red indicates high expression, blue indicates low expression, and the colour bar at the top denotes different clinical stage groupings. (E) Violin plot showing the expression levels of *TNFRSF18* and *CXCL13* in CD8⁺ T cells.

Since we performed scVDJ‐seq, which offers more comprehensive insights into T cells, we prioritized T cells for further in‐depth analysis. To assess T cell phenotypes across distinct clinical stages, we stratified T cells into six groups according to TNM stage and tissue origin: P (Patient 1–6P), T2 (Patient 1–2T), T3 (Patient 3–5T), T4 (Patient 6T), LP (Patient 5LiverP), and LM (Patient 5LiveT) (Figure 1E; Table S1). Furthermore, to be more specific to certain cell subtypes (Figure 2A; Figure S1C,D), annotation was conducted using established marker genes from previously published literature.^39^ Comparative gene expression analysis between tumour and paracancerous tissues showed that CD8⁺ T cells had more differentially expressed genes than CD4⁺ T cells. This suggests that CD8⁺ T cells undergo more extensive functional alterations due to the modulatory effects of the tumour microenvironment.

Based on the above results, we separated the CD8⁺ T cells for further analysis (Figure 2B). Next, we performed differential expression analysis of CD8⁺ T cells according to clinical grouping to explore the changes in gene expression and immune functions from stage T2 to T3 and from T3 to T4. We identified four genes whose expression levels progressively increased with clinical progression (from T2 to T4), including *RPS26*, *CCL3L1*, *CXCL13*, and *CYBA*. In addition, 10 genes showed a gradual decrease in expression as the disease advanced, including *TOB1*, *TUBA1B*, *TUBA1A*, *TNFRSF18*, *PIM1*, *IRF9*, *SF3A1*, *NONO*, and *PHLDA1* (Figure 2C,D). Additionally, we assessed the dynamic alterations in the immune‐related functions of CD8⁺ T cells across various clinical stages through gene set enrichment analysis (GSEA). This analysis identified marked changes in pathways associated with antigen processing, presentation, and immune regulation within the CD8⁺ T cell compartment. Specifically, these functions increased from stage T2 to T3, peaked at T3, and subsequently declined at T4 (Figure S1E,F; Table S3). Overall, these results indicate that both the functional properties and transcriptional profiles of CD8⁺ T cells are progressively and stage‐dependently remodelled throughout colorectal cancer progression.

Next, we examined the expression patterns of the 14 selected candidate genes and observed that only *TNFRSF18* and *CXCL13* were specifically expressed in tumour tissues and exhausted CD8⁺ T cells. Therefore, we focused our further investigations on these two molecules (Figure 2E; Figure S3A). To validate these expression signatures, we obtained previously published publicly accessible single‐cell RNA‐seq data, comprising 15 colorectal cancer (CRC) samples. SingleR was employed for cell type annotation of the public dataset, leveraging our single‐cell dataset as the annotation reference (Figure S2A). Notably, the change in the expression gradient of *TNFRSF18* (which gradually decreases with clinical stage progression) was reproduced in the independent validation cohort, while there was intergroup heterogeneity in the late expression profile of *CXCL13* (Figure S2B). Thus, the expression pattern of *TNFRSF18* in different TNMs suggests its use as a target for early screening of colorectal cancer. In addition, *CD8⁺ T* cells exhibited elevated *TNFRSF18* expression in colorectal cancer samples with liver metastasis, whereas *CXCL13* was higher in CD8⁺ T cells from non‐metastatic colorectal cancer samples (Figure S2B). We additionally examined the expression of *TNFRSF18* and *CXCL13* in colorectal cancer and other tumours through the GEPIA database.^40^ Not only in colorectal cancer (COAD), but *TNFRSF18* and *CXCL13* also have higher expression in tumour tissues across multiple tumour types, including breast cancer (BRCA) and stomach cancer (STAD), indicating that they may serve as a target for a broad range of cancers (Figure S2C,D).

### 3.4 Identification of *TNFRSF18* as a novel biomarker outperforming CXCL13 for exhausted CD8⁺ T cells in CRC

**FIGURE 3:**
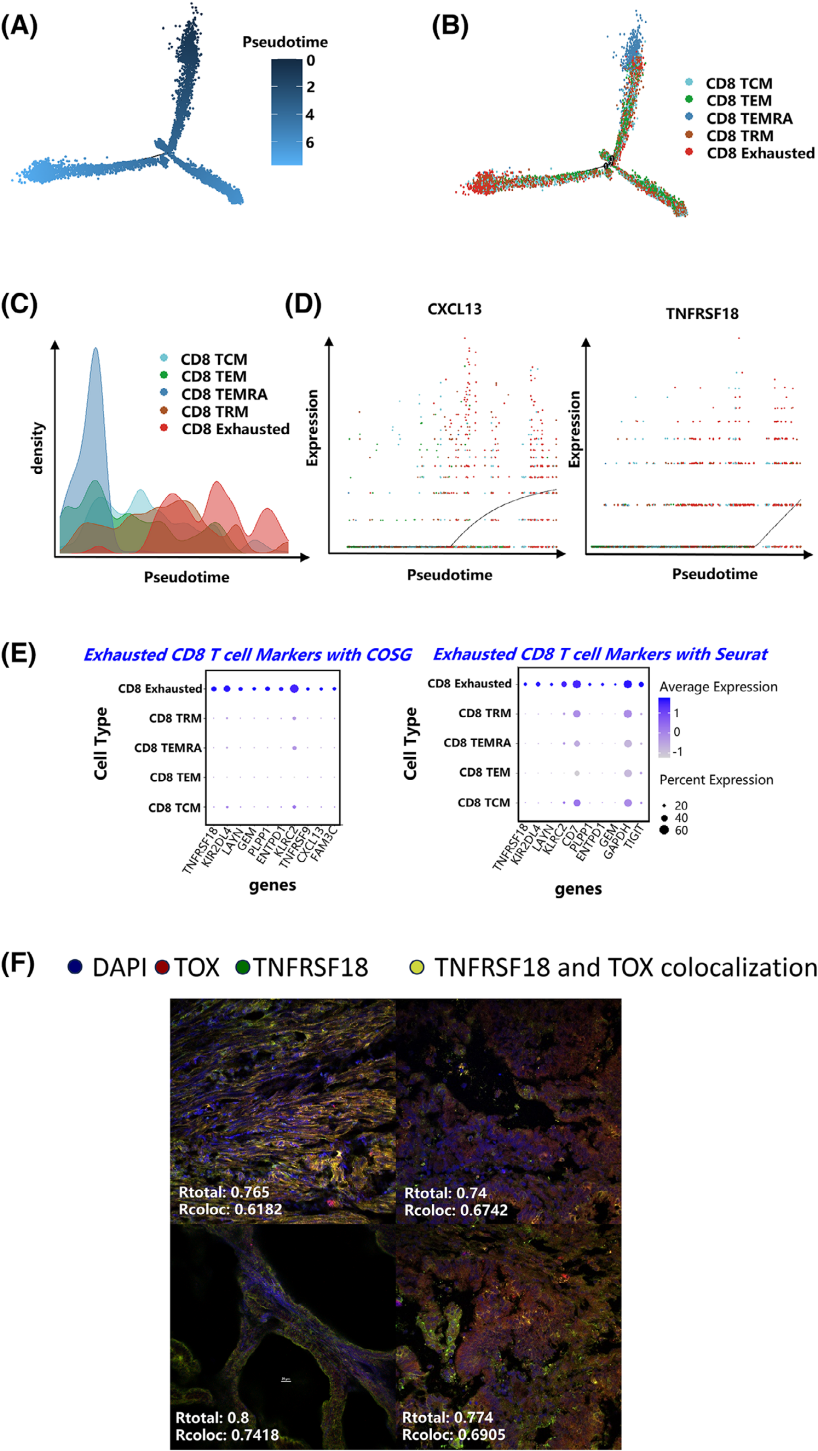
*TNFRSF18* is identified as a novel marker of exhausted CD8⁺ T cells in colorectal cancer progression. (A) Two‐dimensional visualization of the CD8⁺ T‐cell developmental trajectory constructed by Monocle 2 (based on the DDRTree algorithm), with colour indicating the pseudotime calculated by Monocle 2. (B) Cell types overlaid on trajectory as in (A), but with different colours and labels representing cell types, and the black line indicating the inferred cell differentiation trajectory. (C) Cell density plot illustrating the distribution of each cell type along the pseudotime trajectory. (D) Scatter plot showing the dynamic expression changes of *TNFRSF18* and *CXCL13* during cell differentiation (in pseudotime); each dot represents a cell, with colour indicating the cell type. (E) Bubble chart showing the expression of marker genes for exhausted CD8⁺ T (Tex) cells identified by the COSG and Seurat algorithms in CD8⁺ T cells. The size of the dot represents the proportion of cells expressing this gene, and the colour shade represents the average level of expression. (F) Immunofluorescence images (*n* = 3 patients) showing co‐staining of *TNFRSF18* (GITR; green) and *TOX* (red) in colorectal cancer tissues. *DAPI*⁺ nuclei (blue) and areas of colocalization (yellow) are indicated. Scale bar: 20 µm.

Previous single‐cell studies have identified *CXCL13* as a key marker gene for exhausted CD8⁺ T cells.^14^ In our analysis, both *TNFRSF18* and *CXCL13* exhibited markedly higher expression levels in exhausted CD8⁺ T cells from tumour tissues relative to those from adjacent non‐tumorous tissues, indicating that *TNFRSF18* may serve as an additional biomarker for identifying exhausted CD8⁺ T cells in the tumour. Consistently, *TNFRSF18* was robustly recognized as a marker gene for exhausted CD8⁺ T cells using both Seurat's FindMarkers function and COSG analysis.^41^ Additionally, *CXCL13* was also confirmed as a biomarker for exhausted CD8⁺ T cells by COSG (Figure 3E). Furthermore, monocle analysis identified a differentiation trajectory of CD8⁺ T cells (Figure 3A), wherein CD8⁺ TEMRA cells originated early, while exhausted CD8⁺ T cells emerged last (Figure 3B). Expression of both *CXCL13* and *TNFRSF18* was specifically initiated throughout the differentiation process of exhausted CD8⁺ T cells (Figure 3D). Notably, *CXCL13* expression was upregulated earlier in this trajectory, whereas *TNFRSF18* expression appeared at a later stage (Figure 3C,D). Collectively, these results identify *TNFRSF18* as a novel, stage‐specific biomarker for exhausted CD8⁺ T cells in CRC.^42^

The receiver operating characteristic (ROC) curve is a graphical tool utilized to evaluate the balance between sensitivity and specificity in binary classification models over varying threshold values. It is commonly applied in combination with the AUC value for assessing model performance, determining optimal thresholds, and comparing multiple models. A higher AUC value indicates the model's stronger ability to distinguish between positive and negative samples. To further assess the utility of the *TNFRSF18* gene as a potential biomarker for T cell exhaustion, we performed ROC curve analysis on scRNA‐seq data. As presented in Figure S3B, Model C (*TNFRSF18* + *CXCL13*) achieved the highest discriminative ability, followed by Model A (*TNFRSF18*) and Model B (*CXCL13*). Comparative analysis demonstrated that Model C significantly outperformed the other models in distinguishing exhausted from non‐exhausted T cells within CRC tissues. These findings suggest that the combination of *TNFRSF18* and *CXCL13* markedly improves predictive accuracy, as reflected by the notably increased AUC values in the ROC analysis. *TNFRSF18* exhibited superior predictive performance for CD8⁺ T cell exhaustion compared with *CXCL13*. These findings confirm that *TNFRSF18* and *CXCL13* both serve as biomarkers for exhausted CD8⁺ T cells in CRC samples, while *TNFRSF18* exhibits more prominent predictive performance. To ensure the generalizability of *TNFRSF18*, its specific expression in exhausted CD8⁺ T cells was further validated using Seurat's FindMarkers and COSG analysis on an independent public colorectal cancer dataset (*n* = 15) (Figure S3D,E). This validation confirms the reliability of *TNFRSF18* as one marker gene for exhausted T cells in colorectal cancer. Moreover, immunofluorescence co‐localization analysis of patient‐derived CRC tumour sections revealed a significant spatial correlation (Pearson's *r* > .6) between *TNFRSF18* and the canonical CD8⁺ T cell exhaustion marker *TOX*,^43^, ^44^, ^45^ exclusively within the tumour microenvironment (Figure 3F; Table S4; Figure S3C). Collectively, these results identify *TNFRSF18* as a robust and specific biomarker for exhausted CD8⁺ T cells in CRC, outperforming *CXCL13* in predictive accuracy.

### 3.5 TNFRSF18 drives T cell functional exhaustion and alters clonal dynamics

**FIGURE 4:**
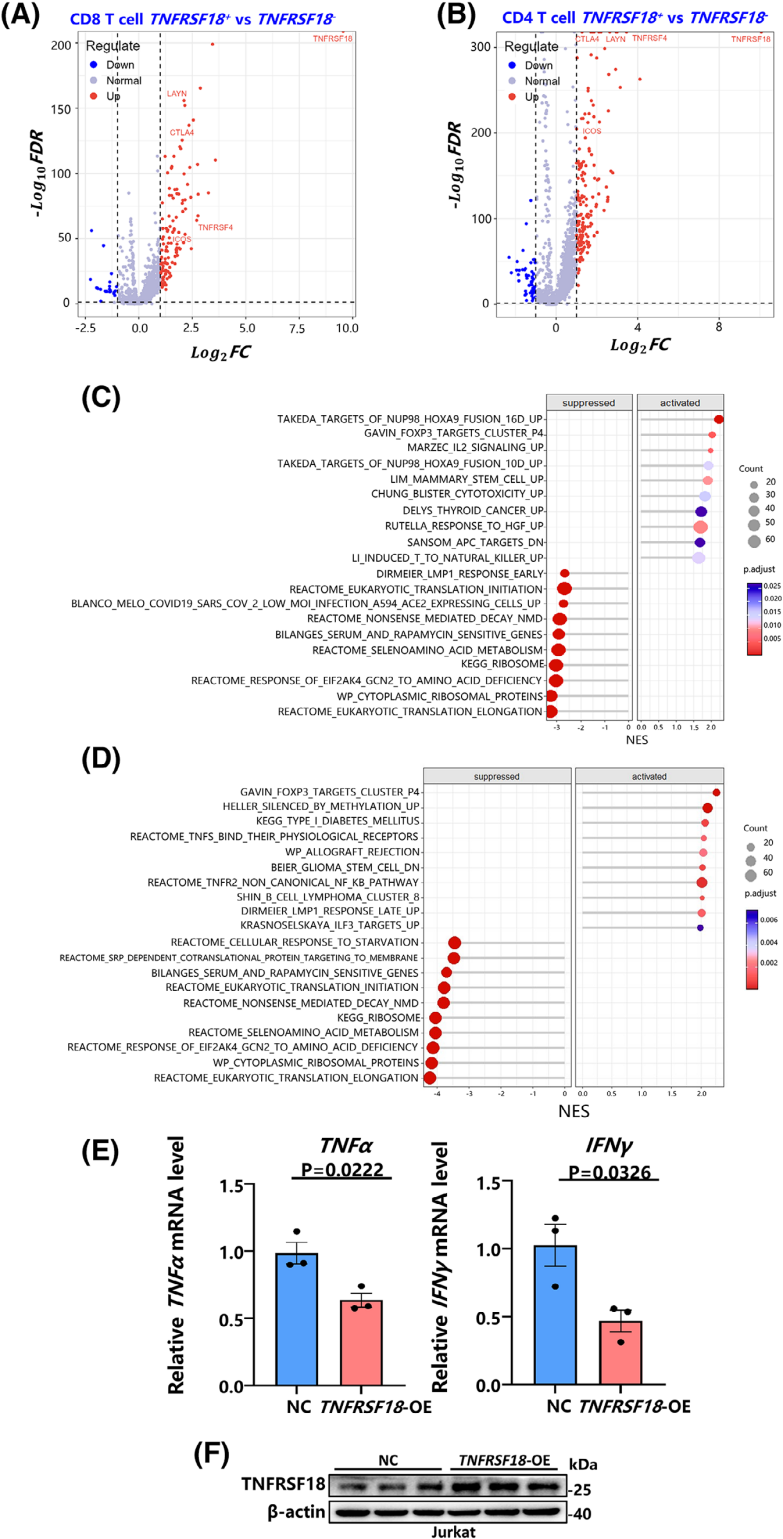
The immune regulatory role of TNFRSF18 in T cells was investigated through differential expression analysis and overexpression experiments. (A) Volcano plots showing the differentially expressed genes (DEGs) between *TNFRSF18*⁺ and. *TNFRSF18* ^−^ CD8⁺ T cells. Significantly upregulated, downregulated, and non‐significant genes are represented by red, blue, and grey dots, respectively. (B) Volcano plots showing the differentially expressed genes (DEGs) between *TNFRSF18*⁺ and. *TNFRSF18* ^−^ CD4⁺ T cells. (C) Lollipop plots depict the significance and enrichment scores of major enriched pathways, along with adjusted p‐values, for DEGs between *TNFRSF18*⁺ and *TNFRSF18* ^−^ groups in CD8⁺ T cells. (D) Lollipop plots depict the significance and enrichment scores of major enriched pathways, along with adjusted *p*‐values, for DEGs between *TNFRSF18*⁺ and *TNFRSF18* ^−^ groups in CD4⁺ T cells. (E) *TNFRSF18* overexpression impairs T cell effector function. qPCR validation of *IFNγ* and *TNFα* in *TNFRSF18*‐overexpressing Jurkat T cells in comparison to that in control groups (*n* = 3 replicates; *p < .05, unpaired *t*‐test). Data normalized to GAPDH (mean ± SEM). (F) Western blot analysis of *TNFRSF18* expression in Jurkat cells. The left panel represents the negative control (NC) group with electroporation only, while the right panel shows the *TNFRSF18* overexpression (*TNFRSF18*‐OE) group following plasmid transfection. Band intensity reflects relative protein expression levels. Each group included three biological replicates (*n* = 3).

Given that *TNFRSF18* functions as a biomarker gene for exhausted CD8⁺ T cells (Figure 3). We next investigated the association of TNFRSF18 expression with T cell functional states and clonal expansion dynamics in colorectal cancer. To explore how TNFRSF18 affects T cell immune function, CD8⁺ T cells were stratified into *TNFRSF18*‐positive and negative groups for differential gene expression analysis. Notably, as shown in Figure 4A,B, both CD8⁺ and CD4⁺ T cells expressing *TNFRSF18* (*TNFRSF18^+^*) exhibited a distinct upregulation of immune regulatory and clonal expansion‐associated genes, including *CTLA4*,^46^ *ICOS*,^47^ *TNFRSF4*,^48^ and *LAYN*,^49^ when compared with their *TNFRSF18* ^−^ counterparts (Log2FC > 1, *p*‐value < .05). Compared with *CXCL13* ^−^ cells, both CD8 and CD4⁺ T cells expressing *CXCL13* exhibited significant upregulation of immune checkpoint molecules,^46^, ^50^ including *CTLA4* and *PDCD1* (Log2FC > 1, *p*‐value < .05) (Figure 4A,B).

Moreover, we performed GSEA on the differentially expressed genes. As shown in Figure 4C, *TNFRSF18*⁺ CD8⁺ T cells demonstrated signatures suggesting transdifferentiation toward NK‐like phenotypes, while *CXCL13*‐positive expression was associated with enhanced binding of TNF and its receptor (Figure S4C). In CD4⁺ T cells, *TNFRSF18*‐positive expression promoted *TNF* binding to *TNFR2* and activated the atypical NF‐κB pathway (Figure 4D), whereas *CXCL13*‐positive expression augmented *PD‐1* signalling (Figure S4D). Both *TNFRSF18* and *CXCL13* are critical regulators of T cell function and influence the TNF signalling pathway (Figure 4; Figure S4, Table S5). We constructed a *TNFRSF18*‐overexpressing Jurkat T cell line and subsequently performed RT‐qPCR assays to evaluate the effect of *TNFRSF18* overexpression on T cell activation in Jurkat cells. As shown in Figure 4E,F, overexpression of *TNFRSF18* significantly decreased the mRNA levels of *TNF‐α* and *IFN‐γ* in Jurkat cells (Table S10), suggesting that *TNFRSF18* suppresses the production of T cells’ effector cytokines, supporting its role in facilitating T cell exhaustion.

Additionally, to explore the impact of *TNFRSF18* and *CXCL13* on T cell clonal expansion, we analyzed single‐cell VDJ‐seq data. As shown in Figure S5, CD8⁺ T cells positive for *TNFRSF18* or *CXCL13* exhibited a higher clonal expansion capacity. In contrast, within CD4⁺ T cells, clonal expansion capacity was higher in cells negative for *TNFRSF18*, and *CXCL13* expression had no significant effect on their clonal expansion (Figure S5, Table S6).

### 3.6 Characterization of the distinct cross‐talk between T cells with malignant tumour cells originating from epithelial cells in CRC

**FIGURE 5:**
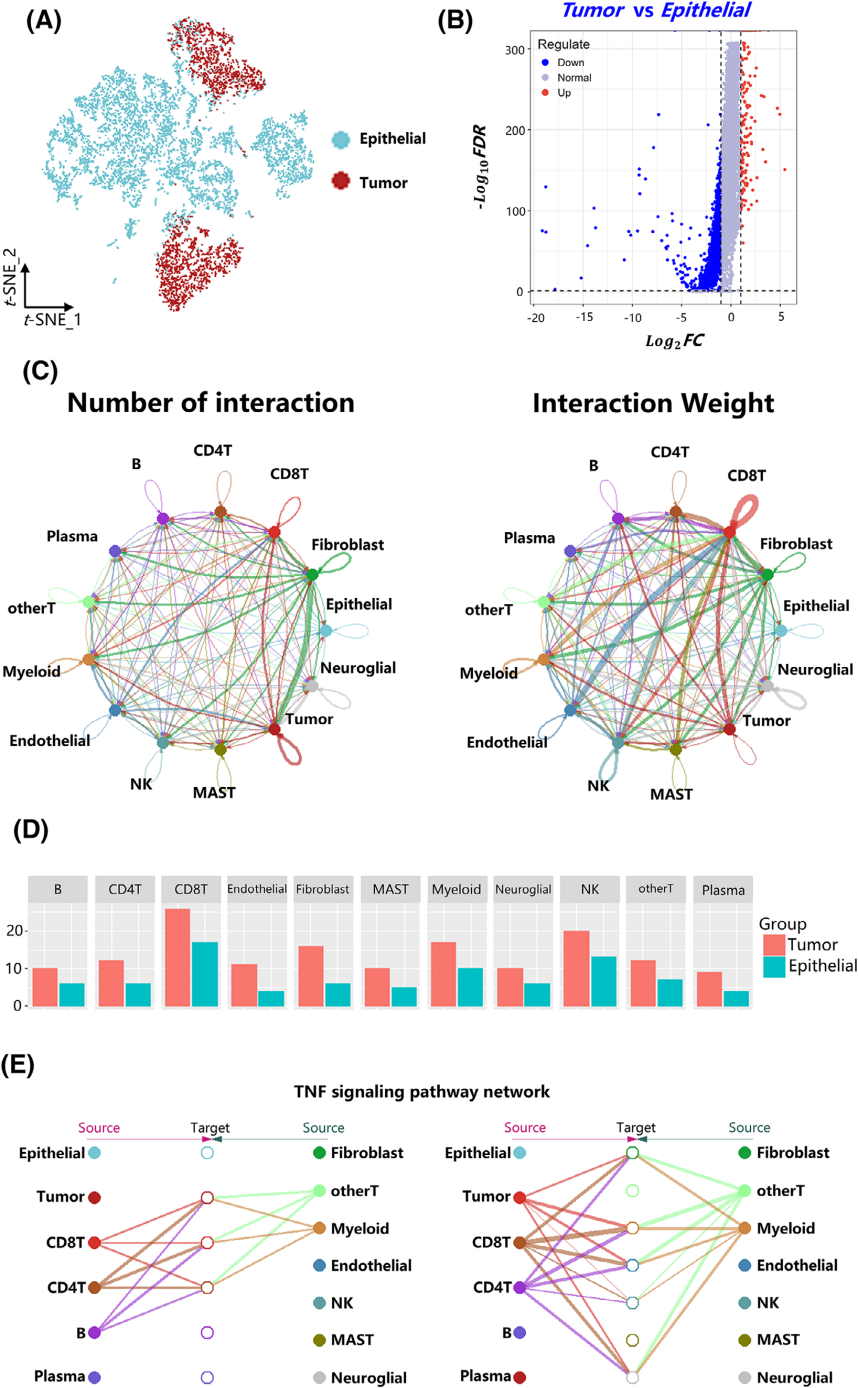
Identification of malignant tumour cells and construction of an intercellular communication network. (A) tSNE plot visualization of different cell types. Each dot denotes an individual cell; the colour denotes cluster origin. (B) Volcano diagram demonstrating differentially expressed genes between tumour cells and epithelial cells. (C) Circle plot illustrating ligand–receptor interactions. The size of each dot represents the number of cells within the corresponding cluster, while the width of the connecting lines indicates the strength of communication between different cell clusters. (D) Bar plot showing the differences in the number of communications between epithelial cells and tumour cells with other cell types. (E) Cell communication network diagram, where the middle column represents the cell types receiving signals, and the sides represent the cell types sending signals. The thickness of the lines indicates the communication strength, with thicker lines representing stronger communication.

In scRNA‐seq data analyses of CRC, malignant cells are predominantly identified within the epithelial cells.^51^ To identify malignant tumour cells within the epithelial cell population, we separated T cells and B cells from the scRNA‐seq data of CRC tumour tissues to provide internal control references. Malignant cells within the epithelial compartment were then identified using the CopyKat algorithm. A total of 2580 malignant tumour cells were detected among the 8961 epithelial cells annotated in the scRNA‐seq dataset (Figure 5A; Figure S6A, Table S7). To investigate the immune regulation between immune cells and malignant tumour cells, we constructed a cell communication network using CellChat. As shown in Figure 5C, tumour cells demonstrated the highest communication activity, with CD8⁺ T cells showing the strongest interaction weight. By ligand–receptor interaction analysis with CellChat, we detected active communication between tumour cells and various immune cell types, including T cells, B cells, plasma cells and myeloid cells, whereas interactions between these immune cells and epithelial cells were comparatively limited. (Figure 5D). Notably, our earlier findings revealed that *TNFRSF18* and *CXCL13* modulate the TNF signalling pathway in T cells (Figure 4; Figure S4). Consistently, TNF signalling emerged as the dominant pathway mediating tumour cell‐immune cell crosstalk, particularly between tumour cells and T cells.^52^ As shown in Figure 5E and Figure S7A, T cells regulated tumour cells selectively through the TNF signalling pathway, while exerting minimal effects on non‐malignant epithelial cells.

Further analysis identified the primary receptor–ligand pairs involved were TNF‐TNFRSF1A and TNF‐TNFRSF1B, with TNF‐TNFRSF1B contributing more prominently to this interaction (Figure S7B). TNF exhibited high expression levels in immune cells, acting as the predominant ligand source. Expression levels of TNFRSF1A and TNFRSF1B did not differ significantly between tumour and epithelial cells (Log2FC < 1) (Figure 5B), suggesting comparable capacities for ligand reception. However, cell–cell communication analysis revealed that immune cells exerted weaker regulatory effects on epithelial cells than on tumour cells, implying the existence of potential mechanisms by which immune cells preferentially recognize and interact with malignant tumour cells.^53^ Moreover, *TNFRSF18* expression in CD4⁺ T cells could modulate TNFRSF1B‐mediated non‐canonical NF‐κB signalling pathway, with both *TNFRSF18* and *CXCL13* expression influencing the binding affinity of *TNF* to its physiological receptors (Figure 4C,D; Figure S4C,D). Collectively, these findings suggest that immune cells may selectively recognize tumour cells through fine‐tuned modulation of TNF signalling dynamics and receptor–ligand interactions.

### 3.7 Elevated tumour stemness mediates TNF signalling‐dependent immune escape in CRC

**FIGURE 6:**
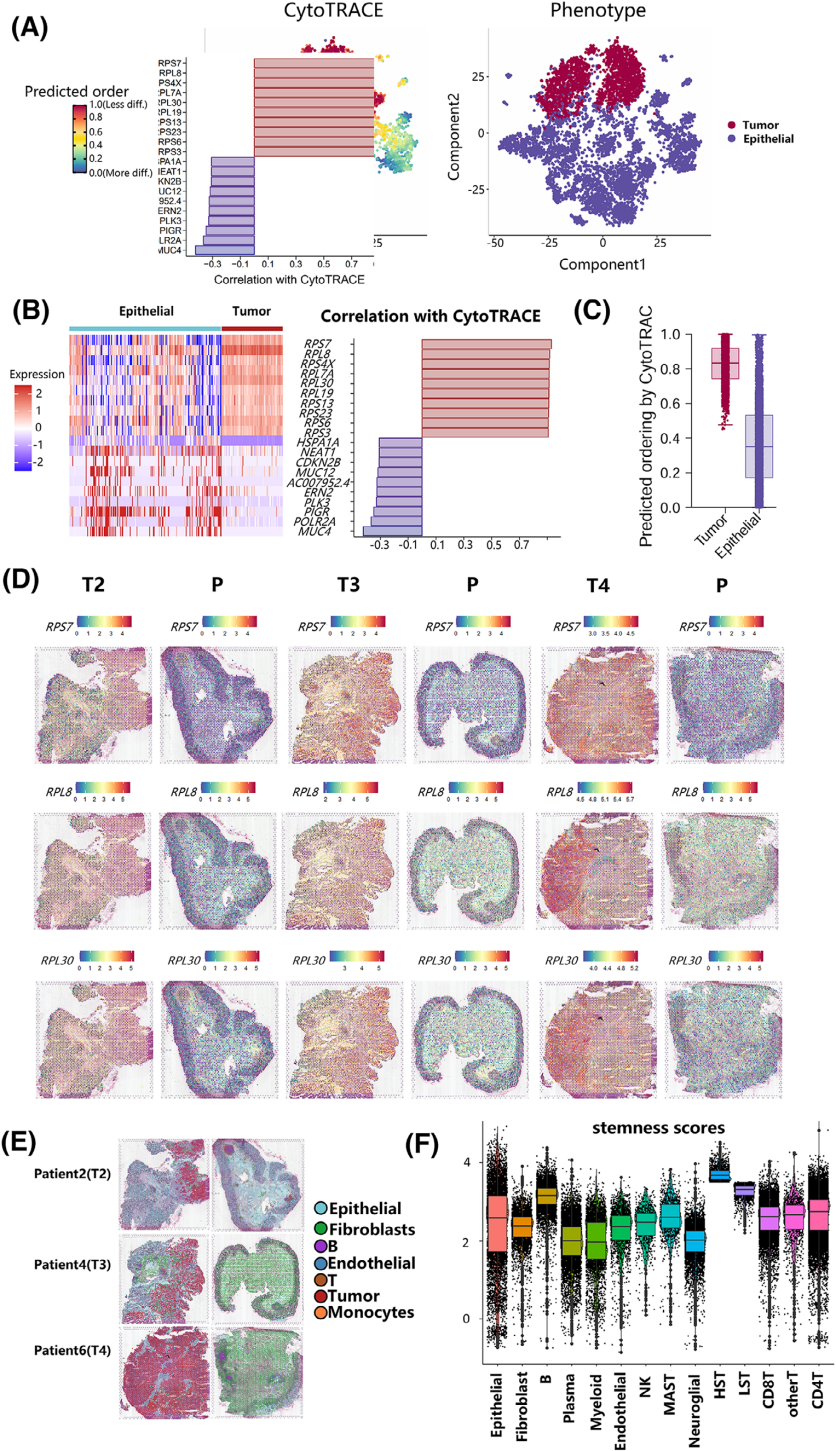
Spatial mapping of stemness signatures across colorectal cancer progression. (A) The results of the CytoTRACE analysis depict the stemness score of each cell. The colour on the left indicates the stemness level, while the colours on the right represent different cell subgroups. (B) Left: The heatmap displays the expression patterns of stemness‐related genes in tumour cells and epithelial cells. Right: Based on CytoTRACE analysis, the top 10 genes showing the strongest positive and negative correlations with the stem cell state were identified. (C) Comparison of CytoTRACE scores between tumour and epithelial cells. Higher scores indicate greater stemness potential. (D) Spatial transcriptomics maps displaying the expression patterns of *RPS7*, *RPL8* and *RPL30* across TNM stages II–IV. Colour intensity indicates expression levels. (E) Cell type localization mapped onto spatial transcriptomics tissue sections using the RCTD algorithm. (F) Violin plots comparing stemness scores across major TME compartments (HST, high stemness tumour cell; LST, low stemness tumour cell).

Given the fundamental role of cellular stemness in cancer progression and therapy resistance, we compared stemness properties between malignant tumour cells and normal epithelial cells.^54^ CytoTRACE analysis revealed the stemness mean score was .83 for tumour cells and .35 for epithelial cells (Figure 6A,C). Genes positively/negatively correlated with stemness were identified (Figure 6B). As shown in Figure 6B and Table S8, genes negatively correlated with stemness were highly expressed in epithelial cells, while genes positively correlated with stemness were significantly upregulated in tumour cells. In particular, three ribosomal genes (*RPS7*, *RPL8*, *RPL30*) met differential expression thresholds (Log2FC > 1, *p* < .05). Analysis of RFS indicated that lower levels of *RPS7*, *RPL8* and *RPL30* expression were linked to improved survival rates (Figure S8B–D), indicating their potential as predictive biomarkers for treatment efficacy.

In order to explore *RPS7*, *RPL8* and *RPL30* causing poor RFS, we constructed a stemness scoring system by the expression of *RPS7*, *RPL8* and *RPL30*. This method generates a stemness score for each cell; higher scores correspond to increased stemness characteristics and an elevated risk of colorectal cancer recurrence. Furthermore, we validated the stemness scoring system by spatial transcriptome data and CRC scRNA‐seq public data, and consistently observed significantly higher stemness scores in malignant tumour cells compared with epithelial cells across all datasets (Figure S9C,E). We classified malignant tumour cells into high grouping (HST, high stemness tumour cells) and low grouping (LST, low stemness tumour cells) using the scoring of malignant tumour cells by the stemness scoring system with a mean value of 3.5 (Figure 6F; Figure S9A,B). Based on this stemness‐based classification, we constructed cell–cell communication networks using CellChat. In the TNF signalling communication network, we observed that high‐stemness tumour cells were less regulated by immune cells, suggesting a potential association between tumour cell stemness and immune evasion, consistent with poor RFS of *RPS7*, *RPL8* and *RPL30* (Figure S8B–D; Figure S9D). Compared with *TNFRSF18*⁺ T cells, *TNFRSF18* ^−^ T cells exhibited stronger cytotoxic activity against tumour cells within the TNF signalling pathway. T cells and other immune cells also induce the apoptosis or immune activation of *TNFRSF18*⁺ T cells through the TNF signalling pathway (Figure S9D). High‐stemness tumour cells exhibited reduced immune regulation and enhanced immune evasion potential, while *TNFRSF18* ^−^ T cells maintained stronger anti‐tumour activity, linking tumour stemness and impaired T cell immunity.

### 3.8 Spatial transcriptomics reveals cell distribution and stemness gene expression

To validate stemness gene expression patterns, we estimate the cell type and status at each point in the spatial transcriptomics (ST) data using the RCTD reverse deconvolution algorithm. ST data were annotated using scRNA‐seq data as reference datasets, identifying seven distinct cell types, the majority of which were epithelial cells (Figure S7D,E). The reverse deconvolution shows the predominant cell type represented in each spot (Figure 6E; Figure S7C,F). In addition, we used ST data from normal epithelial cells of the same patient as a control in CopyKat analysis.^55^ This allowed us to identify malignant tumour cells within the tumour tissue epithelial cell population (Figure 6B–D).

Among the 14 449 epithelial cells in the ST data, 5534 malignant tumour cells were identified (Figure 6E; Table S9). The proportion of tumour cells increased progressively with TNM stage advancement, which was consistent with the corresponding clinical information and validated the reliability of tumour cell identification (Figure 6B–D). In addition, three stemness genes (*RPS7*, *RPL30*, *RPL8*)^56^, ^57^ were confirmed to be highly expressed in tumour tissues. Furthermore, malignant tumour cells within tumour tissues demonstrated elevated expression levels compared with epithelial cells within the tumour tissues (Figure 6D). Spatial transcriptomics analysis further confirmed the differences in tumour cell proportions across various stages and revealed the high expression of stemness genes in tumour tissues and the rationality of the stemness scoring system (which is consistent with cell type and tissues). Additionally, we further explored the spatial transcriptomics data by performing spatial colocalization analysis between the CD8⁺ T cell marker gene (*GZMB*), the macrophage marker gene (*CD68*) and the tumour cell marker gene (*CEACAM5*). This analysis revealed spatial co‐localization of tumour marker with immune cell markers (Figure S10).

Also, the public scRNA‐seq data showed that *RPS7*, *RPL8* and *RPL30* in tumour cells were highly expressed relative to epithelial cells (Log2FC > 1) (Figure S8D). We further examined the expression of *RPS7*, *RPL8* and *RPL30* in other tumours using the GEPIA database. Not only in colorectal cancer (COAD), but *RPS7*, *RPL8* and *RPL30* also have higher expression in tumour tissues in various tumours, such as Thymoma (THYM) and large B‐cell Lymphoma (DLBC) (Figure S8E–G).

**FIGURE 7:**
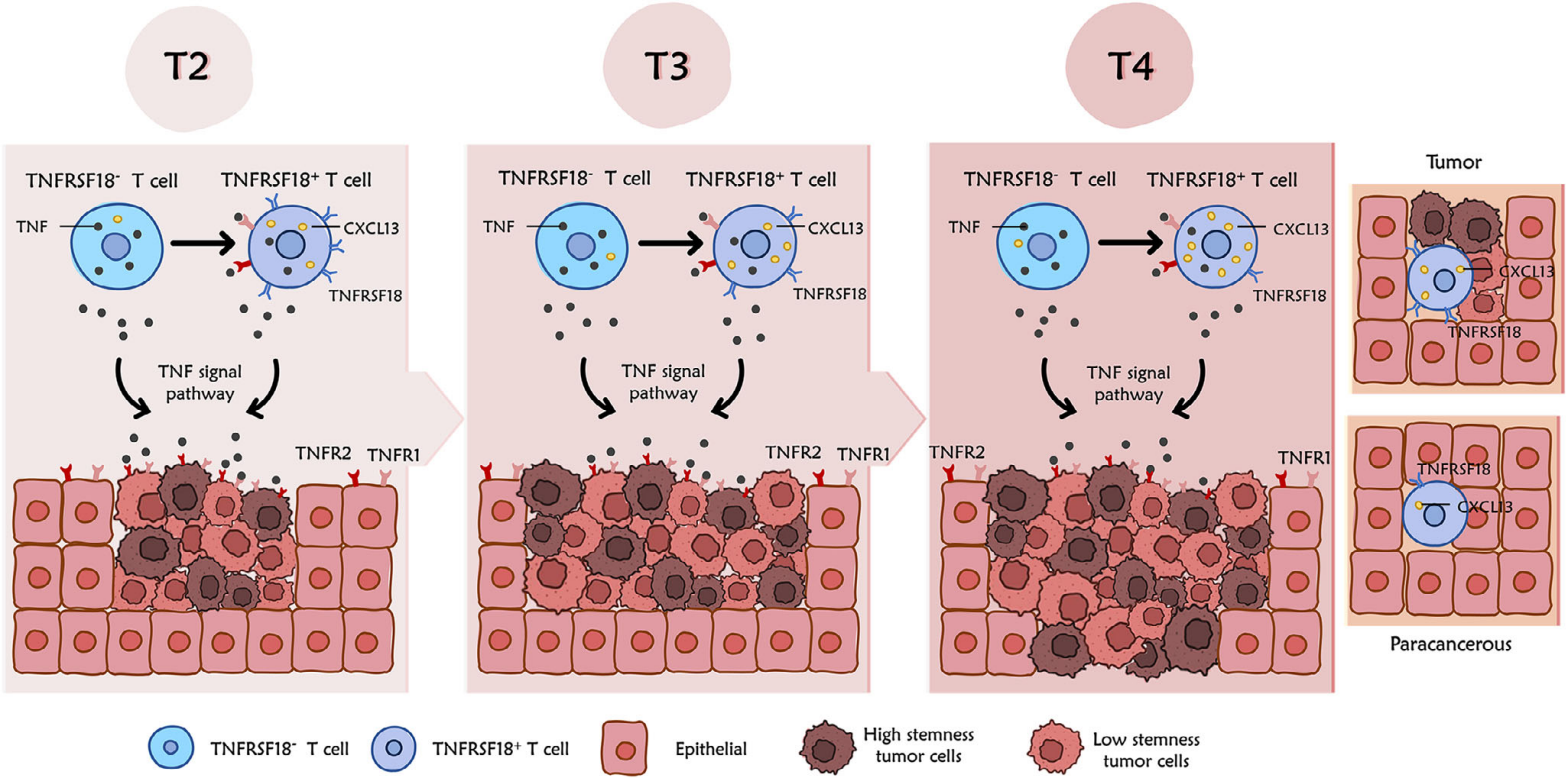
Schematic model for advancing TNM stages shape CD8⁺ T cell exhaustion via *TNFRSF18*/*CXCL13* dynamics and ribosomal stemness in colorectal cancer. This schematic illustrates how advancing TNM stages in colorectal cancer (CRC) shape CD8⁺ T cell exhaustion through divergent *TNFRSF18*/*CXCL13* dynamics and ribosomal stemness. During CRC progression, advancing TNM stages remodel the tumour microenvironment (TME), inducing progressive CD8⁺ T cell exhaustion marked by declining *TNFRSF18* and rising *CXCL13* expression in tumour‐infiltrating T cells despite significantly elevated expression of both markers in tumour compared with paratumour tissues. Concurrently, tumour cells upregulate stemness‐associated ribosomal proteins (*RPS7*, *RPL30* and *RPL8*), enabling evasion of TNF signalling‐mediated immune surveillance. Dysfunction of TNF signalling, due to impaired *TNFRSF18*‐expressing exhausted T cells, fails to suppress high‐stemness tumour cell clusters, creating a feed‐forward loop that promotes immune escape. This *TNFRSF18*‐stemness axis may represent a potential therapeutic target for stage‐specific intervention in CRC.

This study revealed that with advancing TNM staging of colorectal cancer (CRC), *TNFRSF18* expression in CD8⁺ T cells progressively decreased, while *CXCL13* expression increased. Notably, the declining trend of *TNFRSF18* expression was validated in public datasets, suggesting that *TNFRSF18* could act as a promising biomarker for early CRC screening and indicate T cell exhaustion. Mechanistically, *TNFRSF18*⁺ T cells are involved in immune regulation through the TNF signalling pathway, but *TNFRSF18* expression decreased with tumour progression, suggesting that the tumour microenvironment may inhibit the apoptosis and immune activation function of exhausted T cells. Meanwhile, *TNFRSF18* ^−^ T cells have exhibited stronger immunoregulatory activity through the TNF pathway, while tumour cells with high stemness characteristics (high expression of *RPS7*/*RPL30*/*RPL8*) were more resistant to TNF‐mediated immunoregulation. These findings suggest that these stemness‐associated genes represent potential therapeutic targets for selectively eliminating stemness‐high tumour cells in immunotherapy (Figure 7; Figure S9D). Collectively, our integrative analysis of public scRNA‐seq data, GEPIA database, and CRC TNM staging cohorts revealed that tumour cells with high stemness‐related gene expression (*RPS7*, *RPL8* and *RPL30*) resist TNF‐mediated immunoregulation, while dynamic changes in *TNFRSF18* and *CXCL13* expression in CD8⁺ T cells provide promising biomarkers for early CRC screening and monitoring of T cell exhaustion.

## 4 DISCUSSION

### 4.1 During CRC progression, TNM stage remodels tumour environment (TEM), inducing CD8⁺ T cell exhaustion dynamics

CD8⁺ T cells’ functional changes during TNM stage progression were revealed by single‐cell sequencing analysis of tumour samples from different TNM stages (T2, T3, T4) of colorectal cancer. The results revealed that CD8⁺ T cells exhibited progressive immune exhaustion with TNM stage advancement, which was closely associated with dynamic changes in key exhaustion markers (e.g., *CXCL13* and *TNFRSF18*). Specifically, the expression of *TNFRSF18* gradually decreased with the advancing stage, whereas the expression of *CXCL13* increased, suggesting they have a significant impact on T cell function and anti‐tumour immunity. The high expression of *TNFRSF18* in early‐stage (T2) tumours suggests its potential to be a preliminary biomarker for screening of CRC.

These results clarify the role of T cell exhaustion in CRC progression, providing new insights into the molecular mechanisms of clinical staging. In particular, the dynamic expression changes of *TNFRSF18*, as a functional regulator, provide a basis for stage‐stratified immunotherapy.

### 4.2 TNFRSF18 as a superior exhaustion biomarker in tumour‐infiltrating T cells in CRC

In this study, *CXCL13* and *TNFRSF18* were identified as key markers of exhausted CD8⁺ T cells. While *CXCL13* is known to be correlated with the immunosuppressive status of a variety of tumours,^58^, ^59^, ^60^, ^61^ *TNFRSF18* represents a novel validated marker. ROC analyses revealed superior predictive power of TNFRSF18 over CXCL13 for exhaustion, with immunofluorescence validation confirming its reliability. This provides a new molecular target for exhausted T cells’ targeted therapy, and it may be a practical marker for identifying exhausted T cells in single‐cell analysis.

### 4.3 TNFRSF18 modulates T cell dysfunction, enabling immune escape in CRC

*TNFRSF18*⁺ CD8⁺ T cells exhibited upregulated immune checkpoint/regulatory genes and transcriptional features of functional exhaustion. Jurkat T cell overexpression confirmed TNFRSF18's inhibitory role, reducing effector cytokine production in the CRC microenvironment.

scVDJ‐seq revealed enhanced clonal expansion in *TNFRSF18*⁺ CD8⁺ T cells versus *TNFRSF18* ^−^ cells, with inverse patterns in CD4⁺ T cells. These findings demonstrate that TNFRSF18 actively modulates clonal dynamics and promotes immune dysfunction, highlighting its therapeutic potential.

In CRC, TNFRSF18 expression in exhausted CD8⁺ T cells may be crucial for inhibiting apoptosis and maintaining immune activation status. TNFRSF18 expression impaired TNF signalling, weakening T cell cytotoxicity and enabling immune escape of ribosome‐rich tumour clusters.

### 4.4 Ribosomal stemness drives aggressive phenotypes of CRC

Integrated scRNA‐seq and spatial transcriptomics revealed increasing proportions of malignant tumour cells with advancing stages, correlating with poor prognosis. Tumour cells showed elevated stemness‐associated ribosomal genes (*RPS7, RPL8, RPL30*), which serve as diagnostic/prognostic biomarkers and therapeutic targets.

As TNM stages advance, the TME is remodelled, leading to the exhaustion of CD8⁺ T cells, characterized by reduced TNFRSF18 expression and elevated CXCL13 expression. Concurrently, tumour cells elevate stemness‐associated ribosomal proteins (RPS7, RPL8, RPL30), enabling evasion of TNF signalling‐mediated surveillance. This creates a feed‐forward loop: T cells expressing TNFRSF18 exhibited impaired TNF signalling, which compromised their ability to control highly stem‐like tumour cells and consequently accelerated immune evasion. In general, targeting the TNFRSF18–ribosomal axis may offer one potential therapeutic strategy to stage‐specific intervention.

## AUTHOR CONTRIBUTIONS

Hebin Liu conceptualized the study. Hebin Liu and Xiaodong Yang designed the experiments. Tengfei Jia, Xiaojiang Xu, Xiaodong Yang, and Hebin Liu analyzed and interpreted the data. Yingxi Guo conducted the wet‐lab experiments. Xiaodong Yang performed pathological examinations and provided patient tissue samples. Xin meng Cheng and Zeyang Zhou contributed to sample collection. Tengfei Jia and Hebin Liu drafted the manuscript with input from other authors. Hebin Liu and Xiaodong Yang supervised the project and secured finding.

## CONFLICT OF INTEREST STATEMENT

The authors declare no conflict of interest.

## CODE AVAILABILITY STATEMENT

Some of the code used for this subject's raw letter analysis is available from the following website https://github.com/jiatf‐beep/for‐crc‐obj.

## ETHICS STATEMENT

All samples in this study received informed consent from the patients themselves and were approved by the Ethics Committee of the Second Affiliated Hospital of Soochow University (JD‐LK‐2020‐004‐01).

## CONSENT FOR PUBLICATION

All the authors have read and approved the final manuscript.

## Supporting information




**Figure S1**. Cell type annotation results validation, Marker genes and differential expression of CD8⁺ T cells in different clinical stages with GSEA enrichment results. (A) Bar plots showing the proportion of each cell type in individual samples. (B) t‐SNE plots displaying the distribution of cell types based on SingleR automated annotation. (C) Heatmap of characteristic gene expression profiles for CD8⁺ T cell subpopulation annotation. Red represents high expression, and blue represents low expression. The colour bar indicates different CD8⁺ T cell subsets. (D) Heatmap of characteristic gene expression profiles for CD4⁺ T cell subpopulation annotation. (E) GSEA analysis results showing the functional enrichment pathways of differentially expressed genes in CD8⁺ T cells from T2 to T3 stages. (F) GSEA analysis results showing the functional enrichment pathways of differentially expressed genes in CD8⁺ T cells from T3 to T4 stages.

**Figure S2**. Public single‐cell RNA‐seq data cell type annotation and GEPIA database analysis. (A) t‐SNE plot showing the annotation results of T cell subpopulations in a public dataset (GSE132465, *n* = 15). (B) Bubble chart showing the expression of *TNFRSF18* in CD8⁺ T cells from CRC patients at different T/M stages. Dot size indicates the proportion of cells expressing the gene, and colour intensity reflects the average expression level (GSE132465, *n* = 15). (C) Bar plot presenting *TNFRSF18* expression levels in tumour and normal tissues across various cancer types at the RNA‐seq level, based on the GEPIA database. (D) Bar plot presenting *CXCL13* expression levels in tumour and normal tissues across various cancer types at the RNA‐seq level, based on the GEPIA database.

**Figure S3**. ROC curves, immunofluorescence controls, and public data validate the specific expression of *TNFRSF18* in CD8 Exhaustion T cells. (A) t‐SNE visualization of *TNFRSF18* and *CXCL13* expression in CD8⁺ T cells. The gradient colour intensity indicates the gene expression levels. (B) ROC curve analysis comparing the predictive performance of three gene signatures (*TNFRSF18*, *CXCL13*, and *TNFRSF18*+*CXCL13*) for identifying exhausted T cells. The AUC values were .666, .6, and .702, respectively. Model C (*TNFRSF18*+*CXCL13*) exhibited the highest discriminative ability. The X‐axis represents the false positive rate (FPR), and the Y‐axis represents the true positive rate (TPR). A curve closer to the upper left corner indicates better model performance. Model C indicates the combined expression status of both *TNFRSF18* and *CXCL13* genes. (C) Bubble plots showing the expression levels of CD8 Exhausted genes in tumour and paracancerous tissues. (D) Immunofluorescence staining of *TNFRSF18* and *TOX* in colorectal cancer paracancerous tissues (*n* = 3). *DAPI*⁺ nuclei (blue), *TNFRSF18*⁺ (*GITR*; green), *TOX*⁺ (red), and areas of *TNFRSF18* and *TOX* co‐localization (yellow). Scale bars: 20 µm. (E) Bubble plots showing the expression of exhausted CD8⁺ T cell marker genes identified by COSG and Seurat algorithms in CD8⁺ T cells, based on public single‐cell RNA‐seq data (GSE132465, *n* = 15).

**Figure S4**. Differential expression analysis and GSEA enrichment analysis. (A) Volcano plots showing the differentially expressed genes (DEGs) between *CXCL13*⁺ and. *CXCL13* ^−^ CD8⁺ T cells. Significantly upregulated, downregulated, and non‐significant genes are represented by red, blue, and grey dots, respectively. (B) Volcano plots showing the differentially expressed genes (DEGs) between *CXCL13*⁺ and. *CXCL13* ^−^ CD4⁺ T cells. (C) Lollipop plots depict the significance and enrichment scores of major enriched pathways, along with adjusted *p*‐values, for DEGs between *CXCL13*⁺ and *CXCL13* ^−^ groups in CD8⁺ T cells. (D) Lollipop plots depict the significance and enrichment scores of major enriched pathways, along with adjusted *p*‐values, for DEGs between *CXCL13*⁺ and *CXCL13* ^−^ groups in CD4⁺ T cells.

**Figure S5**. Effect of *TNFRSF18* and *CXCL13* expression on clonal expansion. (A–D) Bar plots showing the comparison results from immune repertoire analysis, where CD8⁺ T cells are grouped based on the expression of *TNFRSF18*/*CXCL13*. Different colours represent different levels of proliferation (measured in the number of clones). (E) Bar plot showing the comparison results from immune repertoire analysis, where CD4⁺ T cells are grouped based on the expression of *TNFRSF18*/*CXCL13*.

**Figure S6**. Identification of malignant cells using CopyKAT analysis. (A) CopyKAT analysis based on single‐cell RNA‐seq data. Chromosomal copy number variation (CNV) was inferred to distinguish malignant tumour cells from epithelial cells. The green area represents epithelial cells, and the orange area indicates malignant tumour cells. (B–D) Identification of malignant cells in different spatial transcriptomics samples based on CNV profiles.

**Figure S7**. Cell communication analysis and spatial transcriptome data annotation. (A) Heatmap displaying the interaction strength between different cell types, with darker colours indicating stronger communication intensity.
(B) Bar plot showing the main receptor‐ligand pairs involved in the TNF signalling pathway and their corresponding interaction strength. (C) Representative spatial transcriptomics tissue sections. Six tumour and Paracancerous tissue sections from patients with colorectal cancer at different clinical stages. (D) Histogram showing the number of cells in different cell types in the spatial transcriptome sequencing data. (E) The UMAP plot shows the cell type annotation results in the spatial transcriptome data.
(F) Spatial distribution of different cell types mapped onto the tissue sections.

**Figure S8**. Prognostic analysis of *RPS7*, *RPL8* and *RPL30* and validation of their expression profiles in public datasets. (A–C) Kaplan–Meier survival curves analyzing the association between patient survival and the expression levels of *RPS7*, *RPL8*, and *RPL30*. Red lines represent the high‐expression group, and black lines represent the low‐expression group. (D) Volcano plot displaying differentially expressed genes between tumour cells and epithelial cells based on the public single‐cell RNA‐seq dataset (GSE132465, *n* = 15). (E–G) Bar plots showing the expression levels of *RPS7*, *RPL8* and *RPL30* in tumour and normal tissues across various cancer types at the RNA‐seq level, based on data from the GEPIA database.

**Figure S9**. Constructing and validating the stemness score and building cellular communication networks. (A, B) t‐SNE plot showing the cell type annotation results and stemness score. (C) Violin plot displaying stemness scores across different cell types based on a public dataset (GSE132465, *n* = 15). (D) Heatmap showing the strength of cellular communication between different cell types, with darker colours indicating stronger communication. (E) Spatial transcriptomics stemness score visualization, with colour intensity representing expression levels.

**Figure S10. Co‐localization analysis in spatial transcriptome data**. (A) Spatial co‐localization of the tumour cell marker gene *CEACAM5* with the CD8⁺ T cell marker gene *GZMB*. Red represents *CEACAM5*, green represents *GZMB*, and yellow represents co‐localization. Colour intensity represents the level of expression. (B) Spatial co‐localization of the tumour cell marker gene *CEACAM5* with the Macrophage marker gene *CD68*. Red represents *CEACAM5*, green represents *CD68*, and yellow represents co‐localization.

Supporting Information

Supporting Information

Supporting Information

Supporting Information

Supporting Information

Supporting Information

Supporting Information

Supporting Information

Supporting Information

Supporting Information

## Data Availability

All processed scRNA‐seq and metadata are available in the NCBI Gene Expression Omnibus (GEO) database under the accession code GSE289314. Spatial transcriptome data for this study were accessed under the accession code GSE288119. Other datasets cited are available from the GEO database under the accession codes GSE132465, GSE132257, and GSE144735(https://doi.org/10.1038/s41588‐020‐0636‐z).
