# Supplementary figures and images for: Multi‐omics profiling identifies TNFRSF18 as a novel marker of exhausted CD8⁺ T cells and reveals tumour‐immune dynamics in colorectal cancer
Source: Clin Transl Med. 2025 Aug 6;15(8):e70425. doi: 10.1002/ctm2.70425 (PMC12328248; doi:10.1002/ctm2.70425)

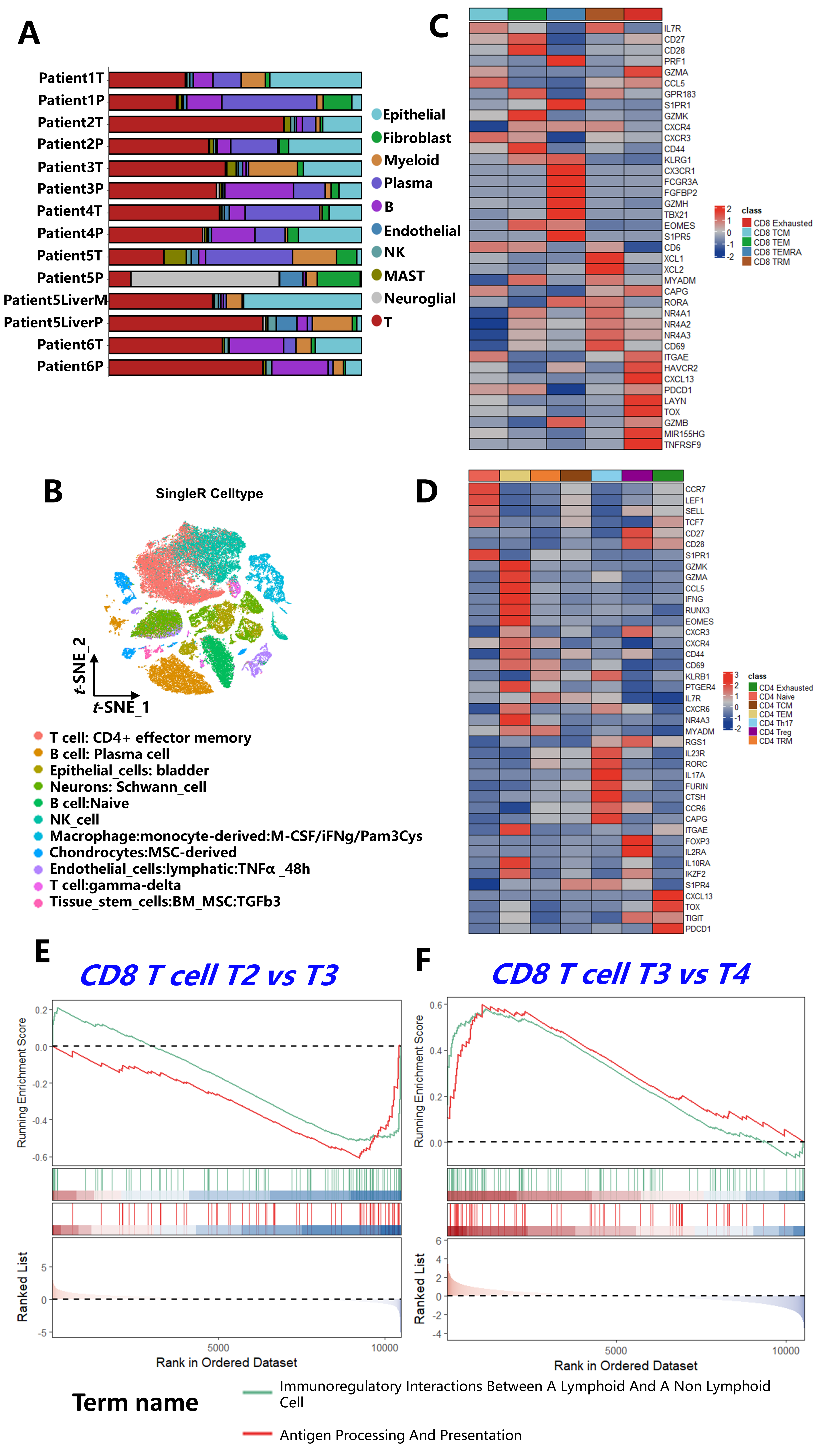

Supplement: Supplementary file 1 — Figure S1. Cell type annotation results validation, Marker genes and differential expression of CD8⁺ T cells in different clinical stages with GSEA enrichment results. (A) Bar plots showing the proportion of each cell type in individual samples. (B) t‐SNE plots displaying the distribution of cell types based on SingleR automated annotation. (C) Heatmap of characteristic gene expression profiles for CD8⁺ T cell subpopulation annotation. Red represents high expression, and blue represents low expression. The colour bar indicates different CD8⁺ T cell subsets. (D) Heatmap of characteristic gene expression profiles for CD4⁺ T cell subpopulation annotation. (E) GSEA analysis results showing the functional enrichment pathways of differentially expressed genes in CD8⁺ T cells from T2 to T3 stages. (F) GSEA analysis results showing the functional enrichment pathways of differentially expressed genes in CD8⁺ T cells from T3 to T4 stages. [file CTM2-15-e70425-s003.TIF]

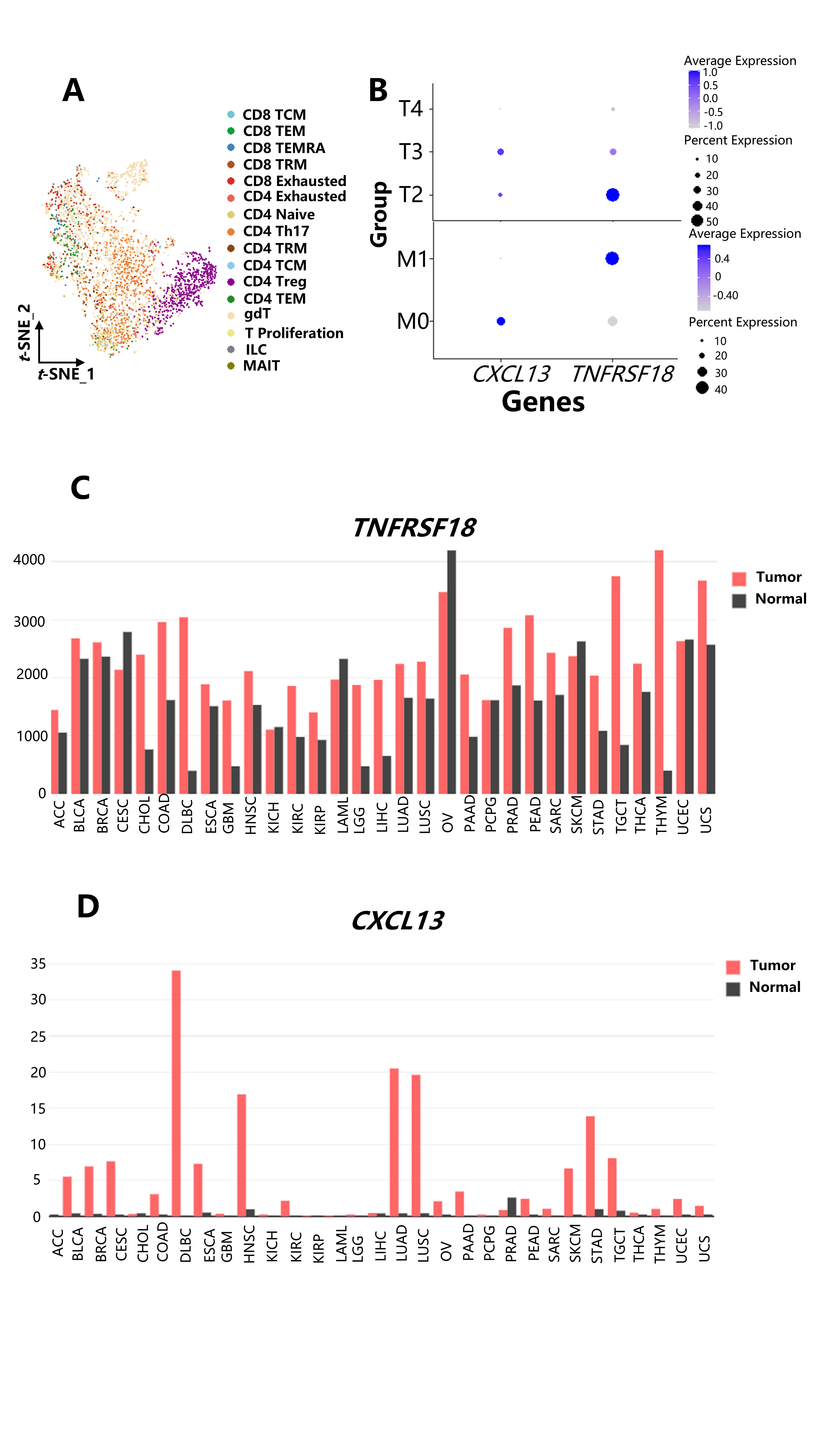

Supplement: Supplementary file 2 — Figure S2. Public single‐cell RNA‐seq data cell type annotation and GEPIA database analysis. (A) t‐SNE plot showing the annotation results of T cell subpopulations in a public dataset (GSE132465, n = 15). (B) Bubble chart showing the expression of TNFRSF18 in CD8⁺ T cells from CRC patients at different T/M stages. Dot size indicates the proportion of cells expressing the gene, and colour intensity reflects the average expression level (GSE132465, n = 15). (C) Bar plot presenting TNFRSF18 expression levels in tumour and normal tissues across various cancer types at the RNA‐seq level, based on the GEPIA database. (D) Bar plot presenting CXCL13 expression levels in tumour and normal tissues across various cancer types at the RNA‐seq level, based on the GEPIA database. [file CTM2-15-e70425-s013.TIF]

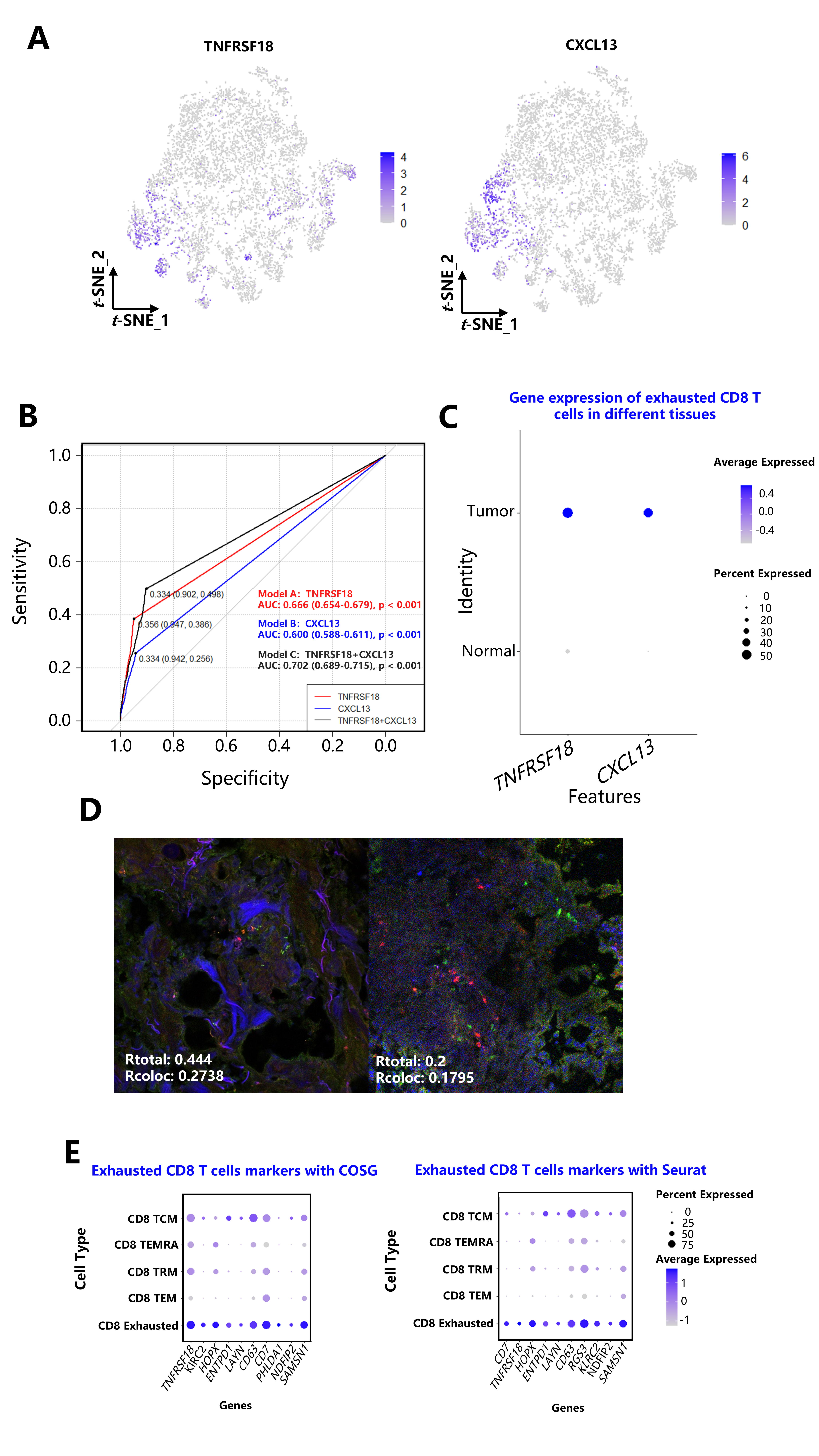

Supplement: Supplementary file 3 — Figure S3. ROC curves, immunofluorescence controls, and public data validate the specific expression of TNFRSF18 in CD8 Exhaustion T cells. (A) t‐SNE visualization of TNFRSF18 and CXCL13 expression in CD8⁺ T cells. The gradient colour intensity indicates the gene expression levels. (B) ROC curve analysis comparing the predictive performance of three gene signatures (TNFRSF18, CXCL13, and TNFRSF18+CXCL13) for identifying exhausted T cells. The AUC values were .666, .6, and .702, respectively. Model C (TNFRSF18+CXCL13) exhibited the highest discriminative ability. The X‐axis represents the false positive rate (FPR), and the Y‐axis represents the true positive rate (TPR). A curve closer to the upper left corner indicates better model performance. Model C indicates the combined expression status of both TNFRSF18 and CXCL13 genes. (C) Bubble plots showing the expression levels of CD8 Exhausted genes in tumour and paracancerous tissues. (D) Immunofluorescence staining of TNFRSF18 and TOX in colorectal cancer paracancerous tissues (n = 3). DAPI⁺ nuclei (blue), TNFRSF18⁺ (GITR; green), TOX⁺ (red), and areas of TNFRSF18 and TOX co‐localization (yellow). Scale bars: 20 µm. (E) Bubble plots showing the expression of exhausted CD8⁺ T cell marker genes identified by COSG and Seurat algorithms in CD8⁺ T cells, based on public single‐cell RNA‐seq data (GSE132465, n = 15). [file CTM2-15-e70425-s012.TIF]

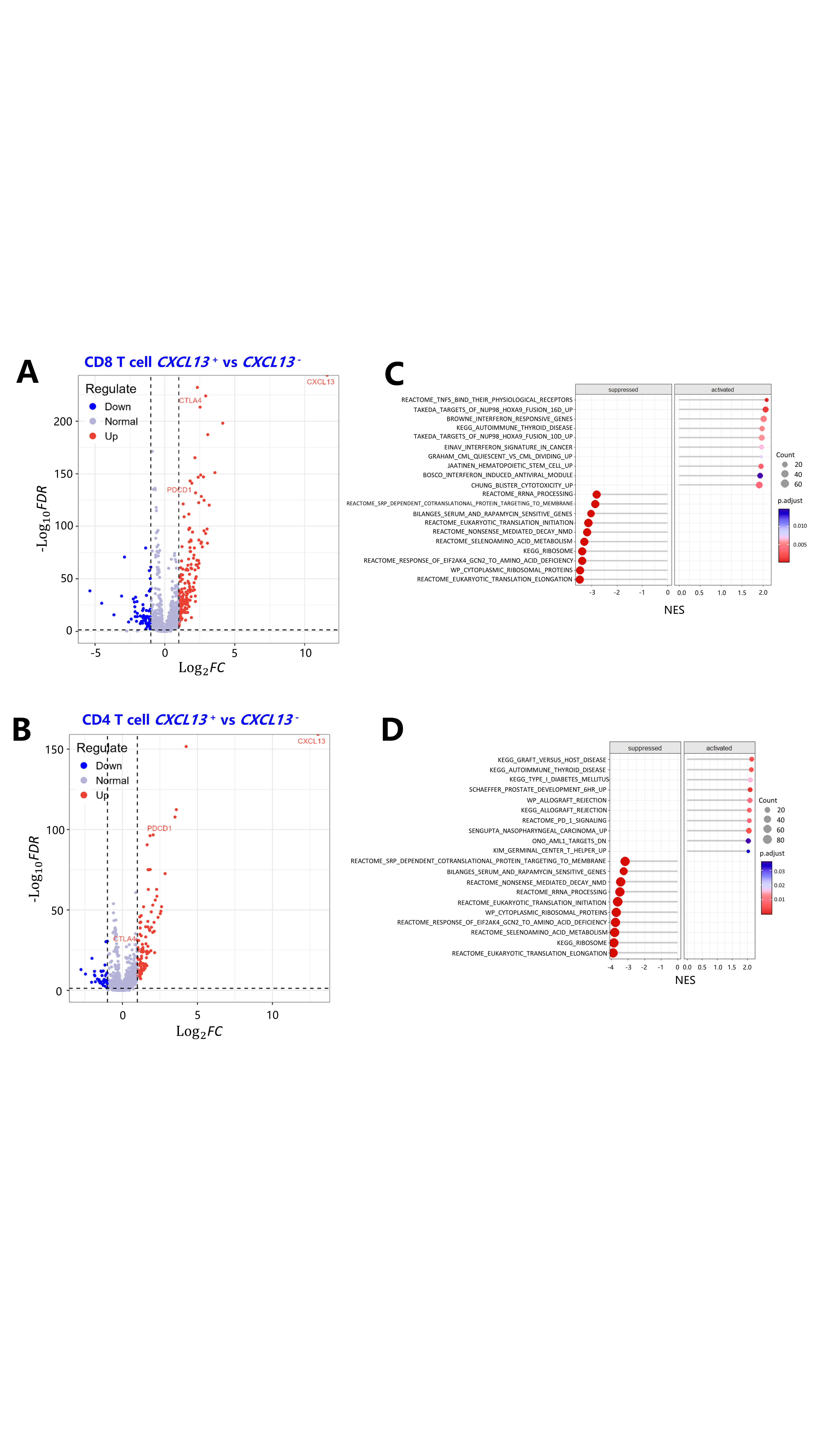

Supplement: Supplementary file 4 — Figure S4. Differential expression analysis and GSEA enrichment analysis. (A) Volcano plots showing the differentially expressed genes (DEGs) between CXCL13⁺ and. CXCL13 − CD8⁺ T cells. Significantly upregulated, downregulated, and non‐significant genes are represented by red, blue, and grey dots, respectively. (B) Volcano plots showing the differentially expressed genes (DEGs) between CXCL13⁺ and. CXCL13 − CD4⁺ T cells. (C) Lollipop plots depict the significance and enrichment scores of major enriched pathways, along with adjusted p‐values, for DEGs between CXCL13⁺ and CXCL13 − groups in CD8⁺ T cells. (D) Lollipop plots depict the significance and enrichment scores of major enriched pathways, along with adjusted p‐values, for DEGs between CXCL13⁺ and CXCL13 − groups in CD4⁺ T cells. [file CTM2-15-e70425-s010.TIF]

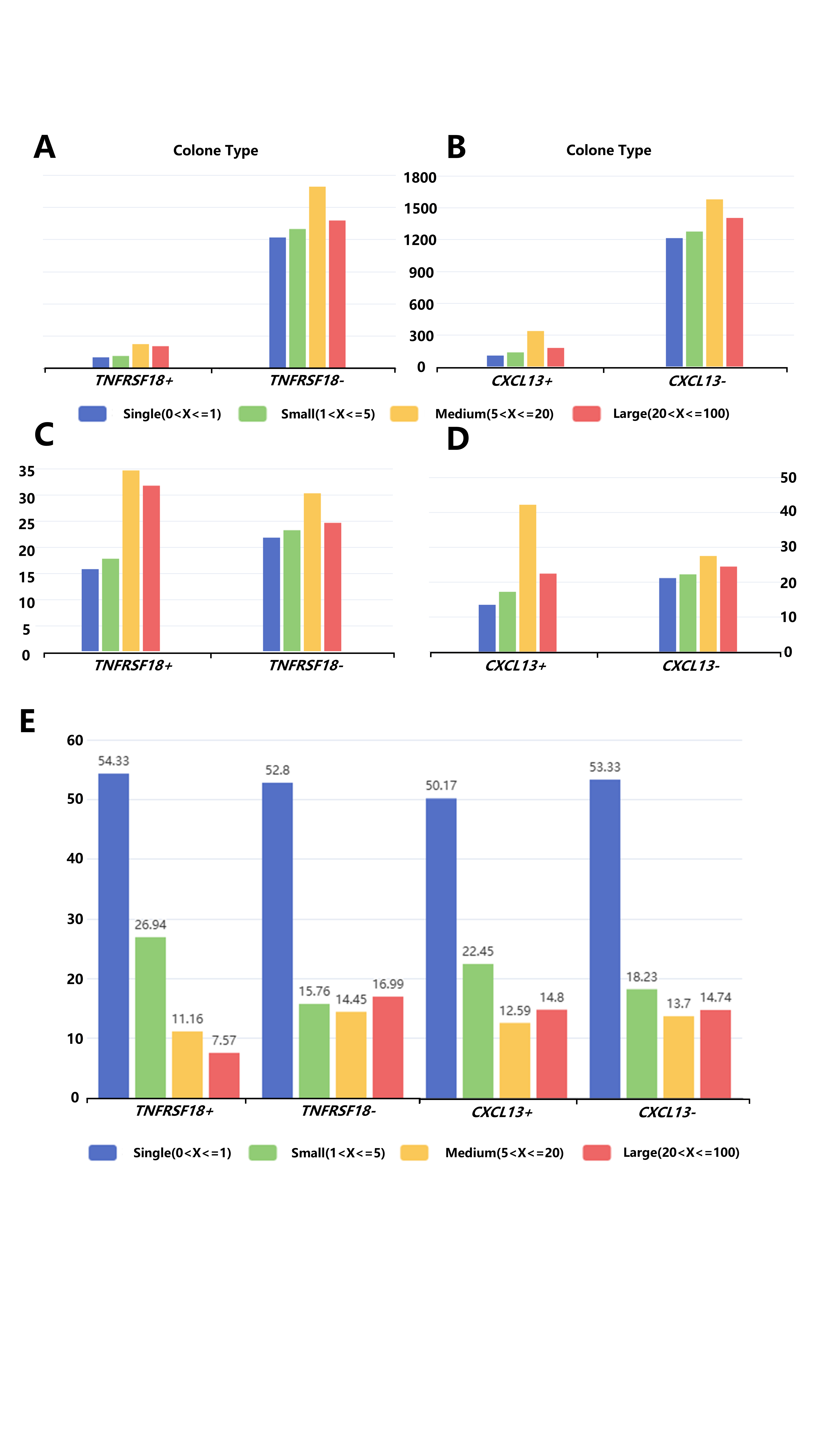

Supplement: Supplementary file 5 — Figure S5. Effect of TNFRSF18 and CXCL13 expression on clonal expansion. (A–D) Bar plots showing the comparison results from immune repertoire analysis, where CD8⁺ T cells are grouped based on the expression of TNFRSF18/CXCL13. Different colours represent different levels of proliferation (measured in the number of clones). (E) Bar plot showing the comparison results from immune repertoire analysis, where CD4⁺ T cells are grouped based on the expression of TNFRSF18/CXCL13. [file CTM2-15-e70425-s008.TIF]

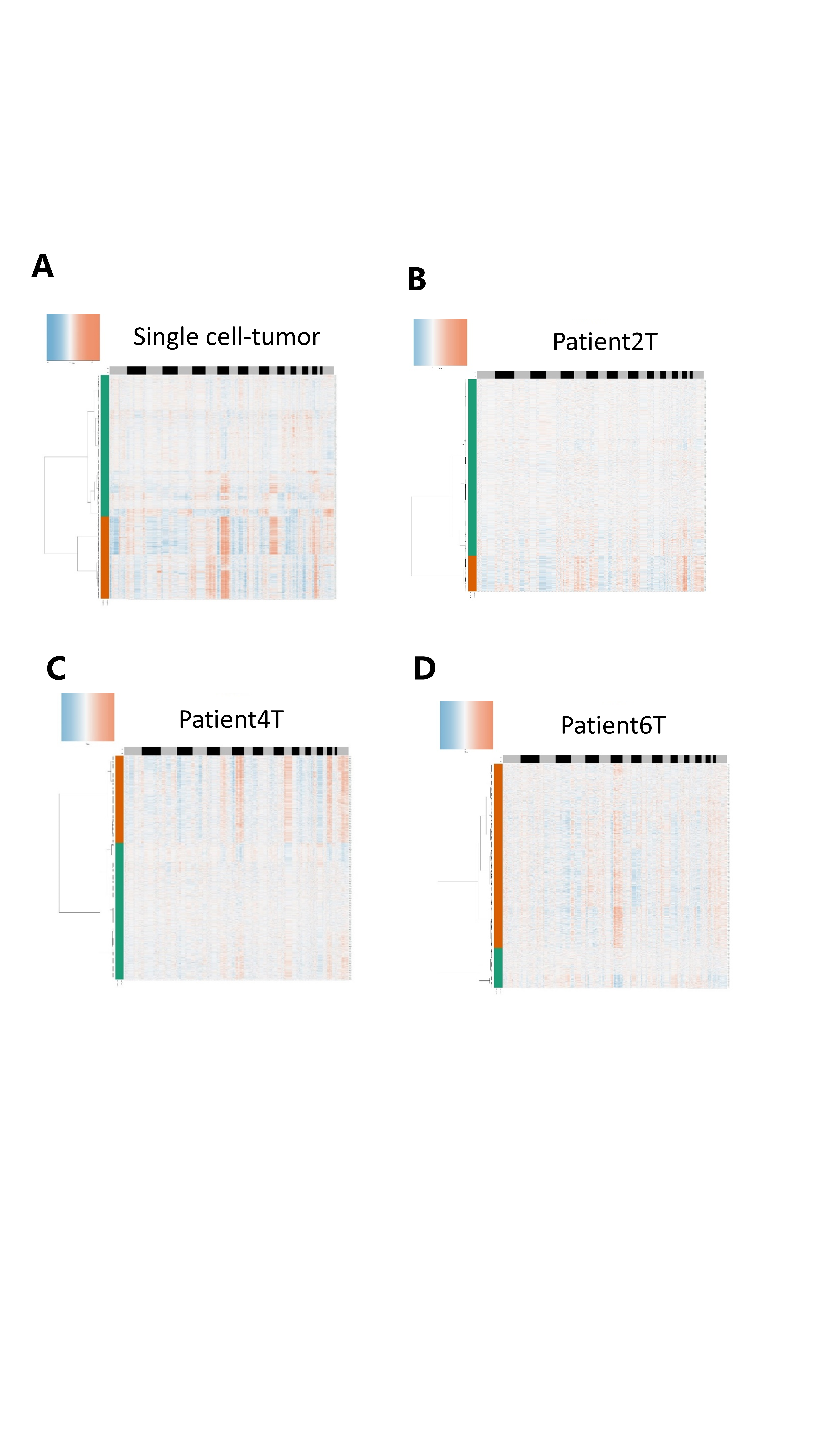

Supplement: Supplementary file 6 — Figure S6. Identification of malignant cells using CopyKAT analysis. (A) CopyKAT analysis based on single‐cell RNA‐seq data. Chromosomal copy number variation (CNV) was inferred to distinguish malignant tumour cells from epithelial cells. The green area represents epithelial cells, and the orange area indicates malignant tumour cells. (B–D) Identification of malignant cells in different spatial transcriptomics samples based on CNV profiles. [file CTM2-15-e70425-s017.TIF]

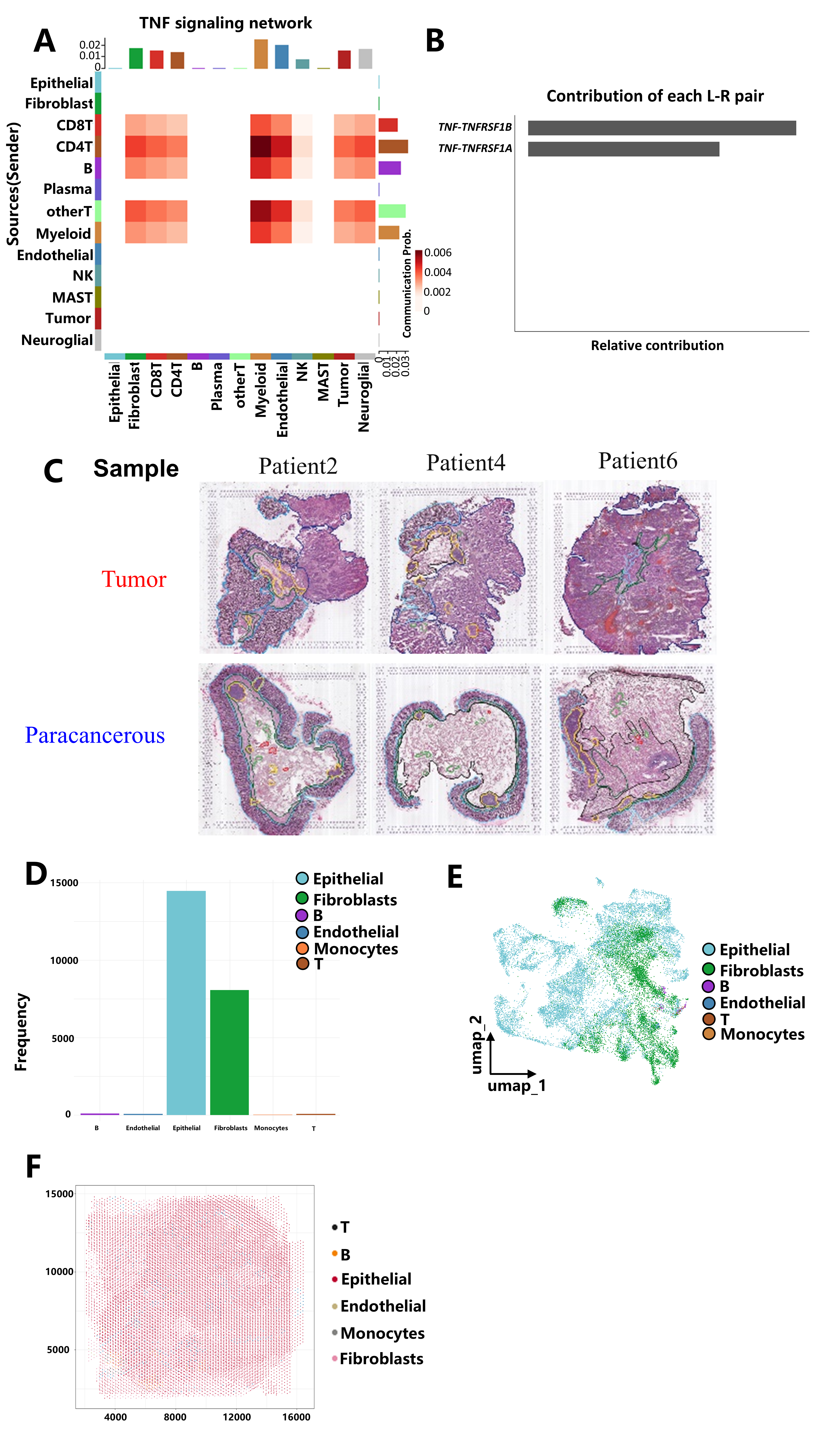

Supplement: Supplementary file 7 — Figure S7. Cell communication analysis and spatial transcriptome data annotation. (A) Heatmap displaying the interaction strength between different cell types, with darker colours indicating stronger communication intensity. (B) Bar plot showing the main receptor‐ligand pairs involved in the TNF signalling pathway and their corresponding interaction strength. (C) Representative spatial transcriptomics tissue sections. Six tumour and Paracancerous tissue sections from patients with colorectal cancer at different clinical stages. (D) Histogram showing the number of cells in different cell types in the spatial transcriptome sequencing data. (E) The UMAP plot shows the cell type annotation results in the spatial transcriptome data. (F) Spatial distribution of different cell types mapped onto the tissue sections. [file CTM2-15-e70425-s020.TIF]

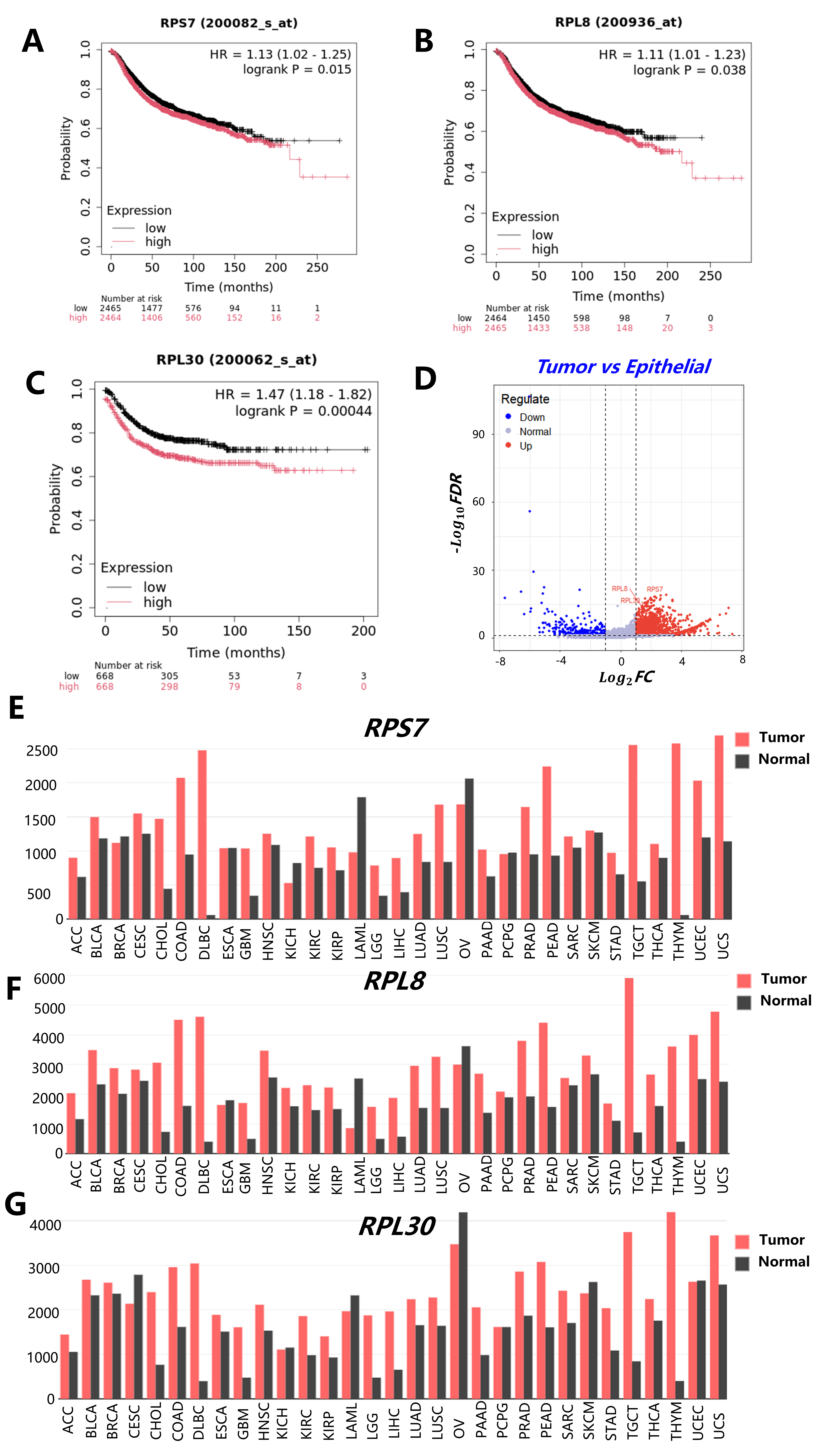

Supplement: Supplementary file 8 — Figure S8. Prognostic analysis of RPS7, RPL8 and RPL30 and validation of their expression profiles in public datasets. (A–C) Kaplan–Meier survival curves analyzing the association between patient survival and the expression levels of RPS7, RPL8, and RPL30. Red lines represent the high‐expression group, and black lines represent the low‐expression group. (D) Volcano plot displaying differentially expressed genes between tumour cells and epithelial cells based on the public single‐cell RNA‐seq dataset (GSE132465, n = 15). (E–G) Bar plots showing the expression levels of RPS7, RPL8 and RPL30 in tumour and normal tissues across various cancer types at the RNA‐seq level, based on data from the GEPIA database. [file CTM2-15-e70425-s004.TIF]

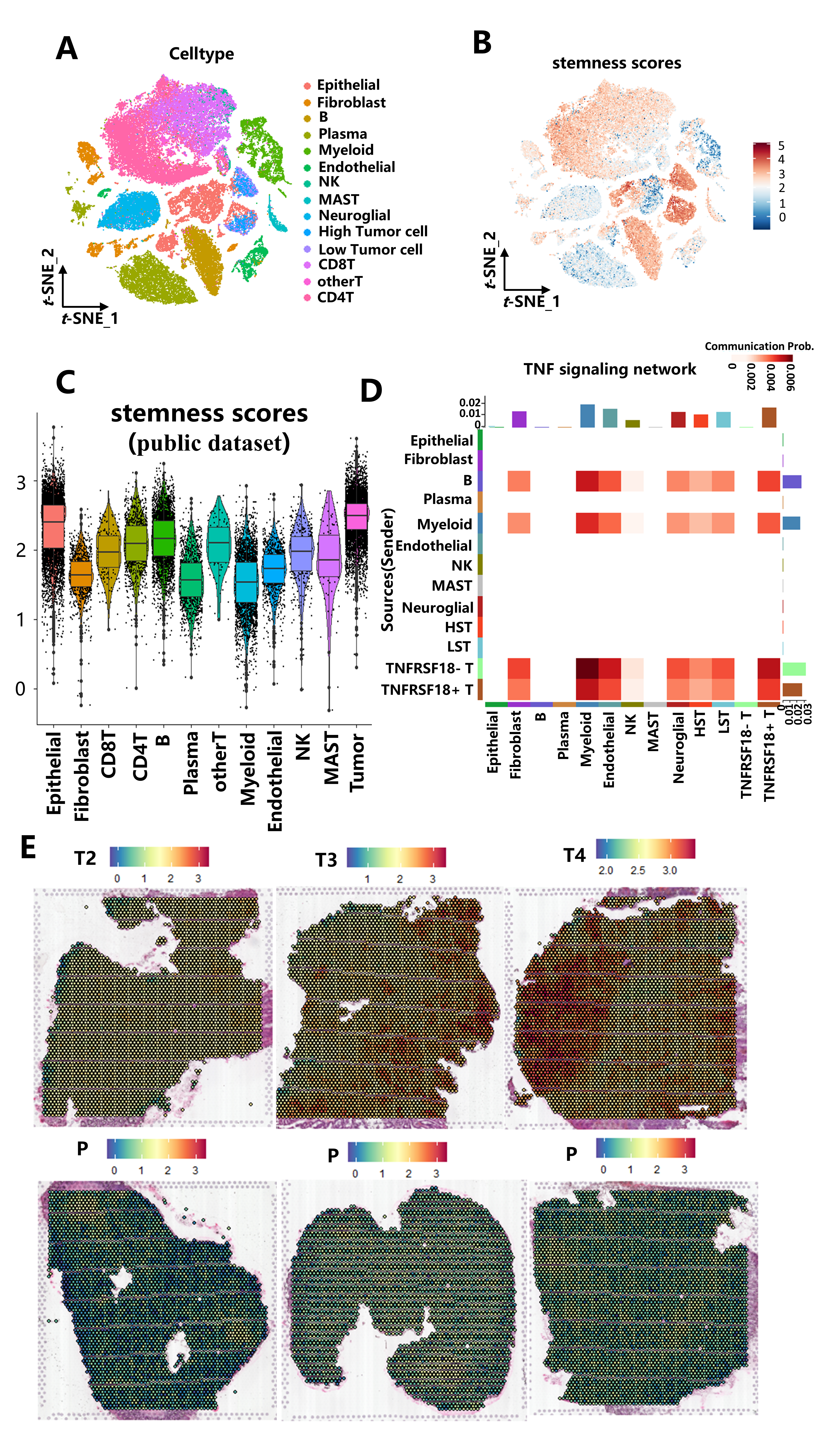

Supplement: Supplementary file 9 — Figure S9. Constructing and validating the stemness score and building cellular communication networks. (A, B) t‐SNE plot showing the cell type annotation results and stemness score. (C) Violin plot displaying stemness scores across different cell types based on a public dataset (GSE132465, n = 15). (D) Heatmap showing the strength of cellular communication between different cell types, with darker colours indicating stronger communication. (E) Spatial transcriptomics stemness score visualization, with colour intensity representing expression levels. [file CTM2-15-e70425-s005.TIF]

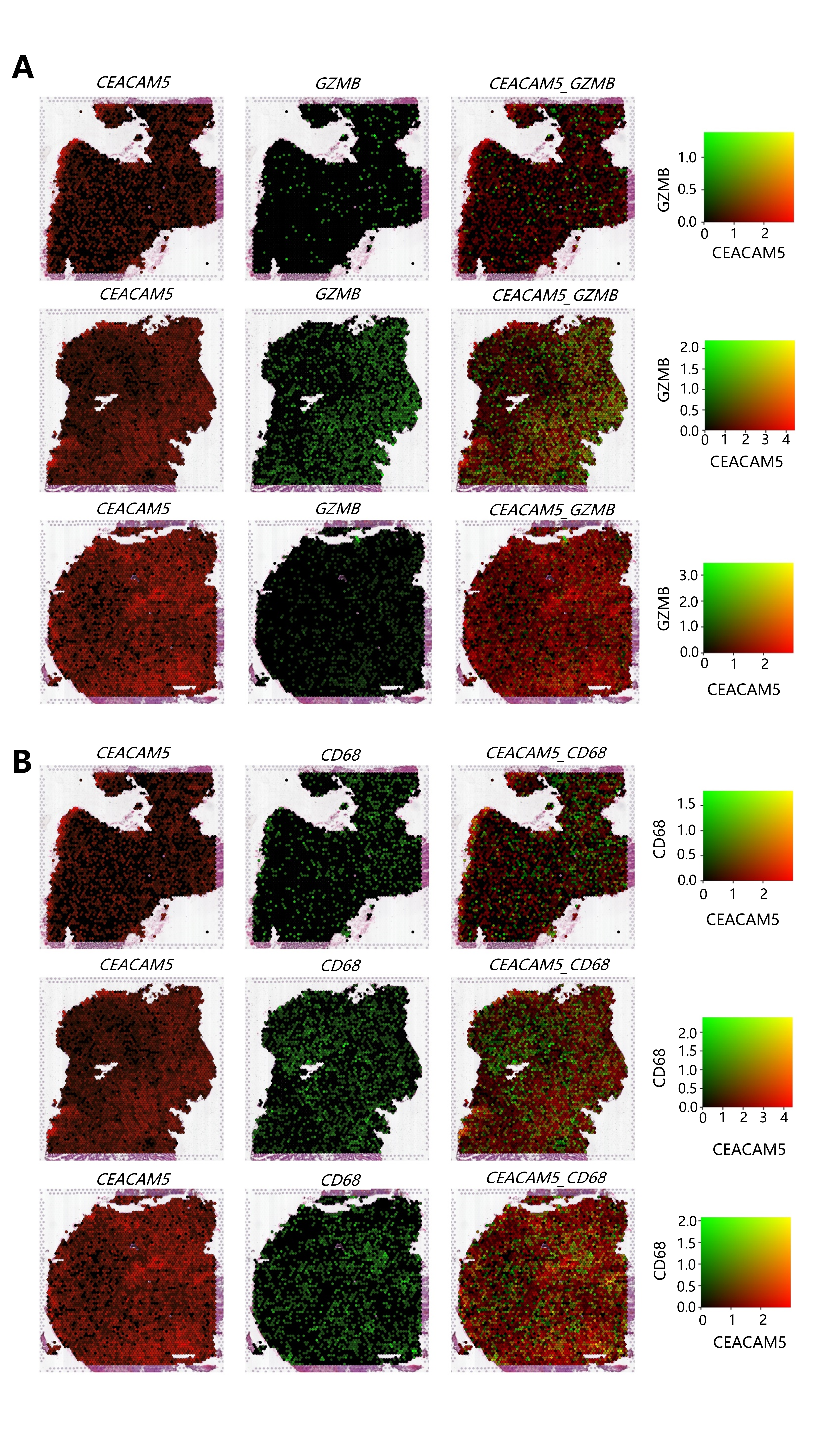

Supplement: Supplementary file 10 — Figure S10. Co‐localization analysis in spatial transcriptome data. (A) Spatial co‐localization of the tumour cell marker gene CEACAM5 with the CD8⁺ T cell marker gene GZMB. Red represents CEACAM5, green represents GZMB, and yellow represents co‐localization. Colour intensity represents the level of expression. (B) Spatial co‐localization of the tumour cell marker gene CEACAM5 with the Macrophage marker gene CD68. Red represents CEACAM5, green represents CD68, and yellow represents co‐localization. [file CTM2-15-e70425-s019.TIF]
